# Comparative analysis of volatile organic compounds for the classification and identification of mycobacterial species

**DOI:** 10.1371/journal.pone.0194348

**Published:** 2018-03-20

**Authors:** Anne Küntzel, Peter Oertel, Sina Fischer, Andreas Bergmann, Phillip Trefz, Jochen Schubert, Wolfram Miekisch, Petra Reinhold, Heike Köhler

**Affiliations:** 1 Institute of Molecular Pathogenesis at the ‘Friedrich-Loeffler-Institut‘ (Federal Research Institute for Animal Health), Jena, Germany; 2 Department of Anaesthesia and Intensive Care, University of Rostock, Rostock, Germany; 3 National Reference Laboratory for Paratuberculosis, Jena, Germany; University of British Columbia, CANADA

## Abstract

**Background:**

Species of Mycobacteriaceae cause serious zoonotic diseases in mammals, for example tuberculosis in humans, dogs, parrots, and elephants (caused by *Mycobacterium tuberculosis*) and in ruminants and humans (caused by *M*. *bovis* and *M*. *caprae*). Pulmonary diseases, lymphadenitis, skin diseases, and disseminated diseases can be caused by non-tuberculous mycobacteria (NTM). Diagnosis and differentiation among *Mycobacterium* species are currently done by culture isolation. The established diagnostic protocols comprise several steps that allow species identification. Detecting volatile organic compounds (VOCs) above bacterial cultures is a promising approach towards accelerating species identification via culture isolation. The aims of this project were to analyse VOCs in the headspace above 13 different species of mycobacteria, to define VOC profiles that are unique for each species, and to compile a set of substances that indicate the presence of growing mycobacteria in general.

**Materials & methods:**

VOCs were measured in the headspace above 17 different mycobacterial strains, all cultivated on Herrold’s Egg Yolk Medium and above pure media slants that served as controls. For pre-concentration of VOCs, needle-trap micro-extraction was employed. Samples were subsequently analysed using gas chromatography-mass spectrometry. All volatiles were identified and calibrated by analysing pure reference substances.

**Results:**

More than 130 VOCs were detected in headspace above mycobacteria-inoculated and control slants. Results confirmed significant VOC emissions above all mycobacterial species that had grown well. Concentration changes were measurable in vials with visually assessed bacterial growth and vials without apparent growth. VOCs above mycobacterial cultures could be grouped into substances that were either higher or equally concentrated, lower or equally concentrated, or both as those above control slants. Hence, we were able to identify 17 substances as potential biomarkers of the presence of growing mycobacteria in general.

**Conclusions:**

This study revealed species-specific VOC profiles for eleven species of mycobacteria that showed visually apparent bacterial growth at the time point of analysis.

## Introduction

About 150 species belong to the family of Mycobacteriaceae. Some members of the *Mycobacterium tuberculosis* complex (MTC) may cause serious zoonotic diseases in mammals, for example *Mycobacterium tuberculosis* causes tuberculosis in humans, dogs, cats, parrots, and elephants [1–5]. Domestic and wild ruminants and swine serve as vectors for *M*. *tuberculosis* and demonstrate asymptomatic infections [6,7]. Tuberculosis in cattle, sheep, and goats is mainly caused by *M*. *bovis* (MB) and *M*. *caprae* [8], but can be transferred to humans, too. With increasing importance, a high number of non-tuberculous mycobacteria (NTM) can cause pulmonary diseases (which resemble tuberculosis), lymphadenitis, skin diseases, and disseminated diseases [9–13]. NTM cover the *M*. *avium* complex (MAC)—which includes *M*. *avium* ssp. *avium* (MAA), *M*. *avium* ssp. *hominissuis* (MAH), *M*. *avium* ssp. *paratuberculosis* (MAP), and *M*. *intracellulare* (MI)—and other NTMs.

The different species of mycobacteria target different, frequently varying hosts. Colonisation often leads to asymptomatic infections, but can also result in clinical disease. Due to their unique cell wall, which consists predominantly of mycolic acids, mycobacteria have a high tenacity. Some species are obligate parasites, while others are found in the environment. So far, little is known about the prevalence of NTM in livestock herds apart from MAP [14]. Paratuberculosis or Johne’s disease is caused by MAP and leads to granulomatous enteritis in ruminants [15,16]. This disease is characterised by intermittently emerging diarrhoea and weight loss. Its enormous economic importance is due to reduced slaughter weight and increased susceptibility to other diseases in infected animals [17]. Paratuberculosis also adversely affects animal’s reproduction [18] and milk yield [19–21]. Because of the high tenacity of MAP in the environment [22] and the incidence in raw milk [23,24], it has frequently been discussed as a pathogen with zoonotic potential [15,25]. There are a few case reports of patients with a suppressed immune system, by human immunodeficiency virus [26] or inflammatory bowel disease [27], who have been tested positive for MAP.

The most sensitive diagnostic method currently available is direct detection of the bacteria via cultural isolation from faeces or tissue samples [28]. Due to the long generation time and high requirements for the media [29], cultivation on solid media is very labour-intensive and time-consuming taking several weeks. After direct detection of bacteria, identifying the species is mandatory [30], for example via polymerase chain reaction (PCR) [31]. Thus far, alternative diagnostic procedures have not proved sufficiently sensitive [32,33], and the labour-intensive procedure cannot be reduced from a two-step to a one-step method [28]. There is an urgent need for an accelerated, sensitive, and specific diagnostic approach.

A potential approach to improve and accelerate the detection of growing bacteria could be the analysis of volatile organic compounds (VOC) released by bacterial cultures [34]. Volatiles are not only emitted from anthropogenic sources, but also from every living cell [35]. With regard to bacterial culture, VOCs can provide information about the presence of growing bacteria and may help to differentiate between bacterial families or even species. This has been shown in a number of studies using different analytical methods: for example, highly selective gas chromatography-mass spectrometry (GC-MS) [36–38] and proton transfer reaction time-of-flight mass spectrometry [39–41] or the less selective simpler techniques such as multi-capillary column–ion mobility spectrometry [42] and differential ion mobility spectrometry [43]. Although these studies have suggested that identification of bacteria and bacterial growth by means of VOC analysis may become feasible [44], only a few studies have targeted VOC profiles from above different species of the same bacterial family, e.g. for Mycobacteriaceae [45–47]. In addition, there are only a few studies that have considered the dependence of VOC profiles on cultivation protocols and conditions [40,48,49].

A lack of knowledge still exists with regard to species-dependent formations of VOCs during bacterial growth of a variety of tuberculous and NTM under standardised conditions of propagation, inoculation, and incubation, as analysed by means of GC-MS.

Therefore, the aims of this study were (i) to prove the presence of growing bacteria of 13 different mycobacterial species by means of VOCs, (ii) to define a core VOC profile for the genus *Mycobacterium*, and (iii) to discriminate mycobacterial species from each other by their VOC profile.

## Material and methods

### Ethics statement

Statements of animal research ethics committees were not required because this study did not include any animal experiment, anaesthesia or necropsy.

Reference strains of bacteria were purchased from the German Collection of Microorganisms and Cell Cultures (DSMZ GmbH, Braunschweig, Germany). Field strains originated from local laboratories for veterinary diagnostic where they had been isolated and cultured before from tissues or faeces of animals in conformity with routine herd diagnostics or animal disease surveillance. All strains were further cultivated according to standard protocols recommended by the National Reference Laboratory for Paratuberculosis.

### Study design

Thirteen species were included in this project (Table 1). All 13 species were cultivated on commercial Herrold’s Egg Yolk Medium (HEYM) containing mycobactin J, amphotericin, nalidixic acid, and vancomycin (Becton Dickinson, Sparks, USA). In total, 140 inoculated vials and 23 control vials were included in this study.

**Table 1:** Study design and included species.

Abbreviation	*Mycobacterium* species / strain	Strain designation	Origin	Inoculum (cfu)	n	Duration of incubation (weeks)
**MB**	*M*. *bovis*	43990	DSMZ GmbH	4.85E+08	3	4
**MAP**	*M*. *avium* ssp. *paratuberculosis*	44133	DSMZ GmbH	6.00E+01	3	4
		04A0386	field isolate from sheep faeces	6.55E+02	3	4
**MAA**	*M*. *avium* ssp. *avium*	44156	DSMZ GmbH	1.55E+08	8	2
		03A2754	field isolate from cattle faeces	3.77E+07	9	2
**MAH**	*M*. *avium* ssp. *hominissuis*	09MA1289	field isolate from swine lymphnode	3.78E+08	9	2
		00A0799	field isolate from cattle lymphnode	2.95E+08	9	2
**MI**	*M*. *intracellulare*	43223	DSMZ GmbH	4.00E+06	9	2
		11MA1917	field isolate from lung tissue of rainbow lorikeet	3.30E+06	9	2
**MT**	*M*. *terrae*	43292	DSMZ GmbH	8.95E+07	9	3
**MM37**	*M*. *marinum*	44344	DSMZ GmbH	9.10E+06	9	3 37°C
**MM30**				9.05E+06	9	3 30°C
**MK**	*M*. *kansasii*	43224	DSMZ GmbH	8.65E+06	9	4
**MC**	*M*. *chelonae*	43804	DSMZ GmbH	8.80E+04	6	2
**MD**	*M*. *diernhoferi*	43524	DSMZ GmbH	8.55E+07	9	2
**MF**	*M*. *fortuitum*	46621	DSMZ GmbH	7.55E+07	9	2
**MP**	*M*. *phlei*	43239	DSMZ GmbH	1.10E+07	9	2
**MS**	*M*. *smegmatis*	43756	DSMZ GmbH	1.28E+07	9	2

cfu–colony forming units; DSMZ GmbH- German Collection of Microorganisms and Cell Cultures, Braunschweig; n–number of replicates.

The isolates were maintained on solid media according to their cultural demands. To prepare the inoculum, three loops of cultured bacteria were added to 10 mL Middlebrook 7H9 liquid medium containing oleic acid, albumin, dextrose, catalase, polymyxin B, amphotericin B, carbenicillin, and trimethoprim (MB-bouillon, produced according to accredited instructions of the National Reference Laboratory for Paratuberculosis). These suspensions were incubated for 7 days at 37°C in an incubator shaker (70 rotations per min) in the presence of sterile glass beads, except one flask containing *M*. *marinum* that was incubated at 30°C (MM30). The bacterial suspensions were thoroughly vortexed and diluted with MB-Bouillon to an optical density of 0.306 ± 0.02. Subsequently, nine replicates per strain were generated by inoculating 100 μl of the bacterial suspension onto each of nine HEYM slants. The vials were sealed with Silicone/Teflon septa and incubated at the appropriate temperature (see Table 1), in a horizontal position for one week and then further in an upright position. Sampling was performed after two weeks of incubation for fast-growing, three weeks for intermediate-growing, and four weeks for slow-growing mycobacteria (Table 1). MM30 was incubated at 30°C due to its adaptation to fish and reptiles [50] and also at 37°C to be able to compare results to the other species. Vials inoculated with 100 μl MB-Bouillon instead of the bacterial suspension served as controls and were also incubated for two, three, and four weeks. This enabled analysis of and correction for media-derived VOCs.

Bacterial growth was visually assessed at regular intervals until the time of analysis, when it was scored as follows:

0.5 points less than 20 colonies apparent on the slant

1 point between 20 and 50 colonies apparent on the slant

2 points between 51 and 100 colonies apparent on the slant

3 points over 100 colonies apparent on the slant or a thin layer of growth

4 points a loosened layer of comprehensive growth is apparent on the slant

5 points a concluded layer of comprehensive growth is apparent on the slant

### Sampling protocol for VOC analysis

Pre-concentration of VOCs from the headspace above the inoculated slants and the pure media control slants were carried out by means of needle trap micro-extraction (NTME), as described by Trefz et al. 2013 [51]. The triple-bed needle trap devices (NTDs, Shinwa Ltd., Japan) were packed with divinylbenzene (DVB, 80/100 mesh, 1cm), Carbopack X (60/80 mesh, 1 cm), and Carboxen 1000 (60/80 mesh, 1 cm). Before first use NTDs were conditioned in a heating device (PAS Technology Deutschland GmbH, Magdala, Germany) at 250°C for at least 12 h under permanent helium flow (1.5 bar), and re-conditioned at 250°C for 30 min before being applied for pre-concentration of the samples. Immediately before sampling all vials were warmed up in a heating block at 37°C (Unitek, Germany) for 20 min. Needles were pierced through the septum, and 20 ml of headspace was bi-directionally passed through the needle by inflating and releasing a 1 ml disposable syringe (Transcoject GmbH, Neumünster, Germany). Each NTD was sealed using a Teflon cap (Shinwa LTD., Japan/PAS Technology Deutschland GmbH, Magdala, Germany) before and immediately after collecting a gaseous sample. In parallel to this procedure, further NTDs were exposed to laboratory room air (n = 10) to be able to estimate unwanted contaminations of the pre-concentration devices during routine handling.

### Identification and quantification of substances

VOC analyses were performed by means of GC-MS. VOCs that thermally desorbed from NTDs were separated by gas chromatography (Agilent 7890A) and detected by mass spectrometry (Agilent 5975C inert XL MSD). For all experiments, a RTX-624 (60 m; 0.32 mm; 1.8 μm film thickness) capillary column from Restek (Bad Soden, Germany) was used. Inlet temperature for desorption was 250°C and the column temperature program for separation worked as follows—40°C for 5 min, 8 K/min to 120°C for 2 min, 10 K/min to 220°C, 20 K/min to 250°C for 4 min. Electron ionisation (70 eV at 250°C) and total ion chromatogram measurements (scan range, 35–250 amu) were applied for all samples. This process has been previously described [51,52]. VOCs were initially identified by a mass spectral library search (NIST 2005 Gatesburg, PA, USA). Analysis of pure reference substances (origin of chemicals in S1 Table) and comparison of GC retention times and mass of all selected marker substances specified subsequent identification and quantification.

For NTME calibration, a liquid calibration unit (LCU, Ionicon Analytik GmbH, Innsbruck, Austria) provided humidified standards of pure references in different concentration levels. The signal-to-noise ratio was used to calculate the substance limit of detection (LOD, signal-to-noise ratio 3:1) and limit of quantification (LOQ, signal-to-noise ratio 10:1). Noise was determined from blank samples (n = 10). VOC concentrations under LOD were set to zero. Supplement S2 Table provides methodological details of identified substances (retention time and quantitative parameters, such as LOD and LOQ).

### Selection of VOCs

NTME GC-MS analysis resulted in more than 130 individual volatile substances detected in the headspace of vials and quantified by analysis and calibration of pure reference substances (section 2.3). Values represent the concentrations of the volatile compounds in the headspace of the vials. We compared the VOC concentrations above control slants, above mycobacterial cultures, and from the laboratory room air. This was done in order to differentiate between VOCs originating from the material or the media, those arising from or being consumed by bacterial cultures, and those existing in the surrounding air of the laboratory. The inclusion criterion for a volatile was that its concentration was above that of the surrounding laboratory room air. VOCs that had a higher concentration in the laboratory air or had a high variability above the control slants were excluded.

### Statistical analysis

Concentration values of selected VOCs of all 23 control vials and 140 inoculated vials were included in the statistical data analyses. R x64 (version 3.3.1, R Development Core Team, New Zealand) in conjunction with R studio (version 0.99.903, R-Tools Technology Inc., Canada) and Microsoft Excel 2016 (Microsoft Corporation, USA) were used. Numerical data are presented as medians and percentiles (25–75%). To identify significant differences between groups of data, the Kruskal-Wallis Test followed by the Tukey HSD-test was applied. The Mann-Whitney-U-Test was employed to identify VOCs with significant inter-strain variability in concentration. Values with p < 0.05 were considered statistically significant. For visualisation, a three-coloured heat map with normalised values was prepared. A principal component analysis (PCA) was used to convert possible correlated variables into components with the objective of visualising those components and aligning them with different qualities of the samples: classification of mycobacteria, the colony forming units (cfu) of the inoculum, visually assessed bacterial growth, and duration of incubation.

## Results

### Detecting the presence of growing bacteria via VOCs

**Fig 1:**
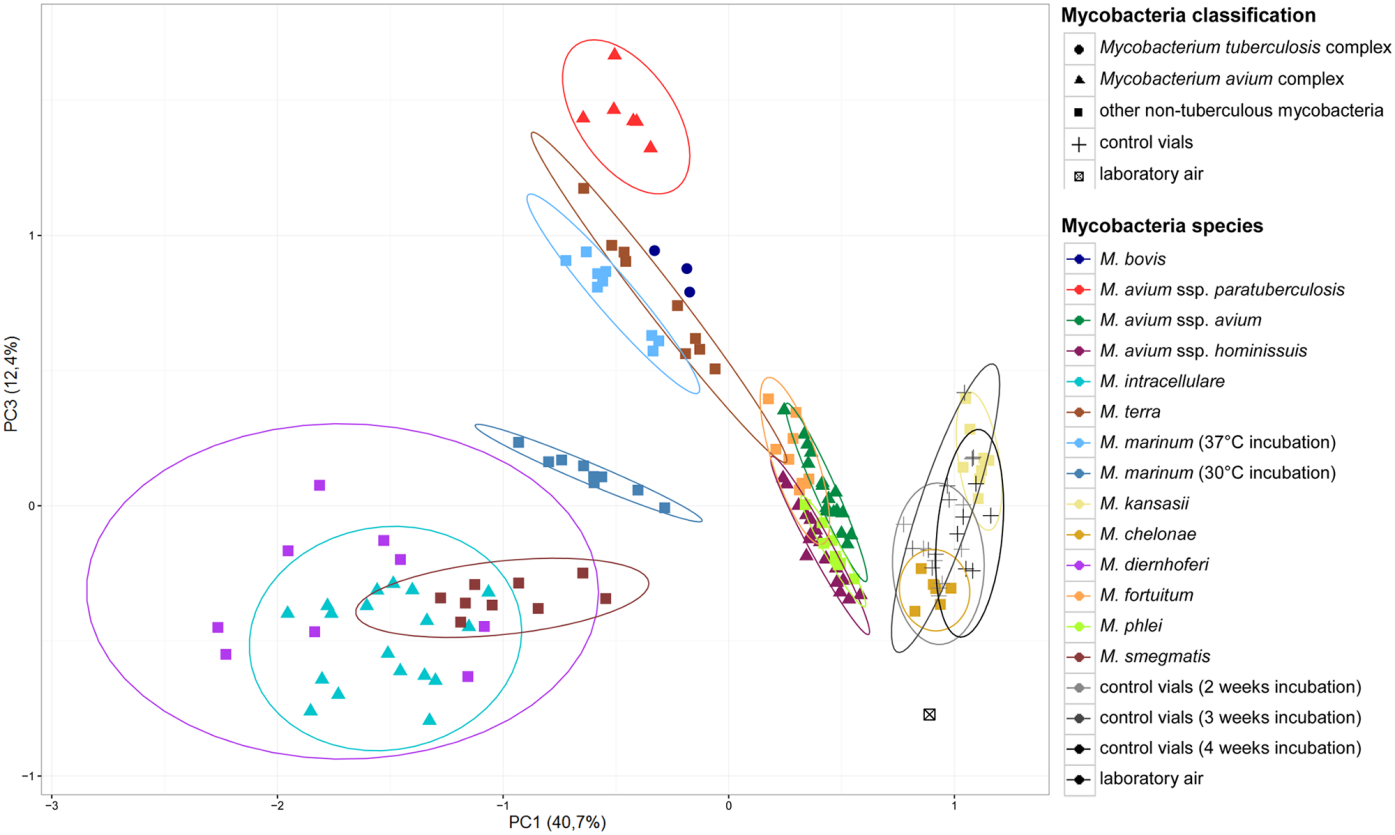
Differentiation of mycobacterial species by VOC profile. Illustration is based on a principal component analysis (PCA).

The VOC composition in the headspace above slants clearly distinguished between control vials and vials with bacterial growth (Fig 1).

**Fig 2:**
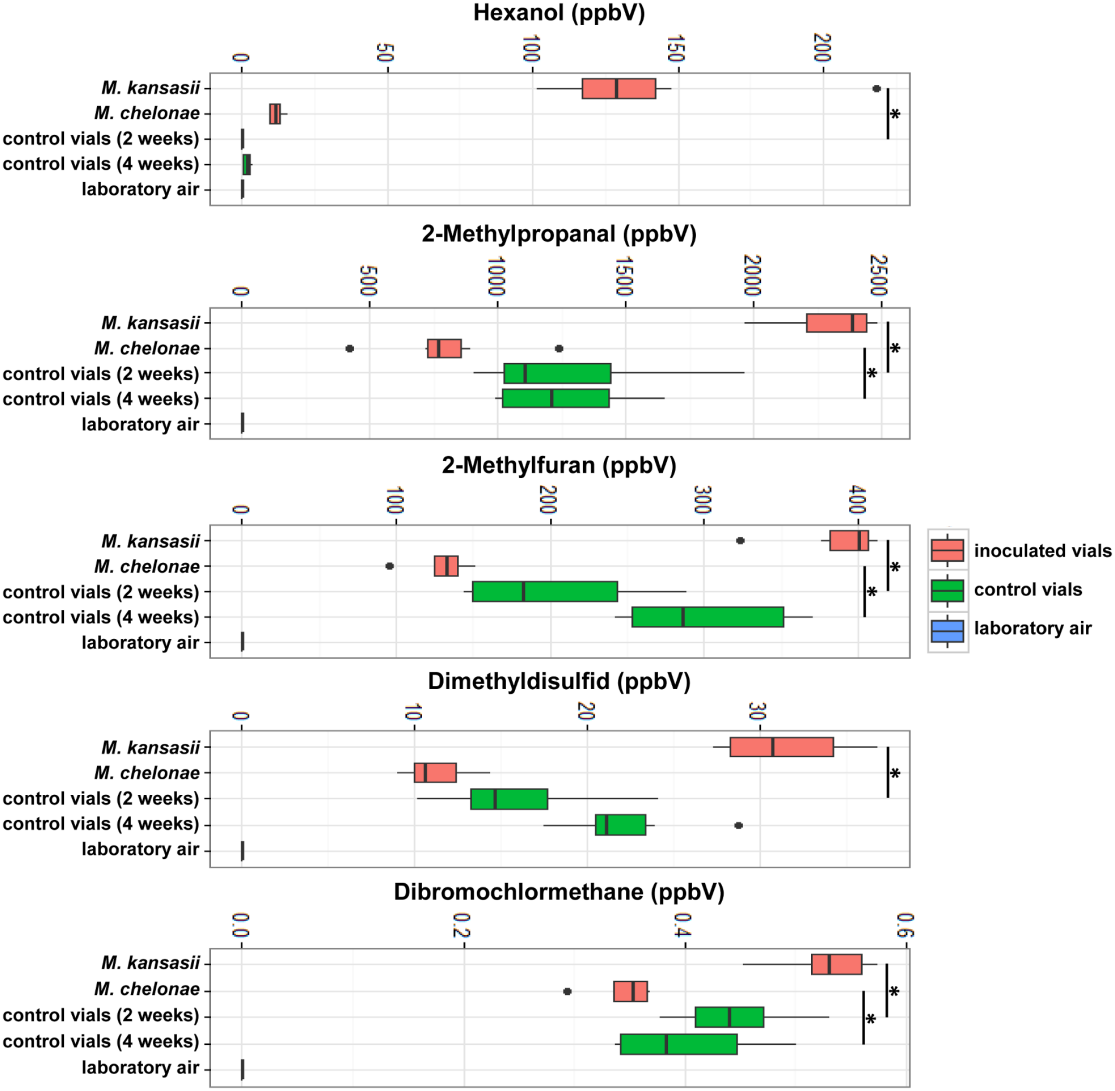
Significant differences in VOC concentration above vials with poor bacterial growth at the time point of analysis and above non-inoculated control vials. *—significant when p-value < 0.05.

Four species of mycobacteria (i.e. MAP, MB, MT, MM30, and MM37), formed well-defined clouds in a PCA (Fig 1), which separated them from all other species. The clusters of MS, MI, and MD overlapped, as did MF, MAA, MAH, MP (abbreviations in Table 1). The same visualisation with a PCA, but grouped via different qualities of the samples shows that the clusters do not resemble colony forming units (cfu) of the inoculum, bacterial growth or duration of incubation. An exception was the VOC compositions above inoculated slants with poor bacterial growth, i.e. MK and MC (Fig 1), because these vials presented themselves in the same cluster as the control vials. Hence, they were excluded from further investigations regarding species-specific VOC profiles and comparison of those. However, they did have significant differences in the concentration of 14 VOCs in the case of MK and six VOCs in the case of MC when compared to control vials (Fig 2).

**Fig 3:**
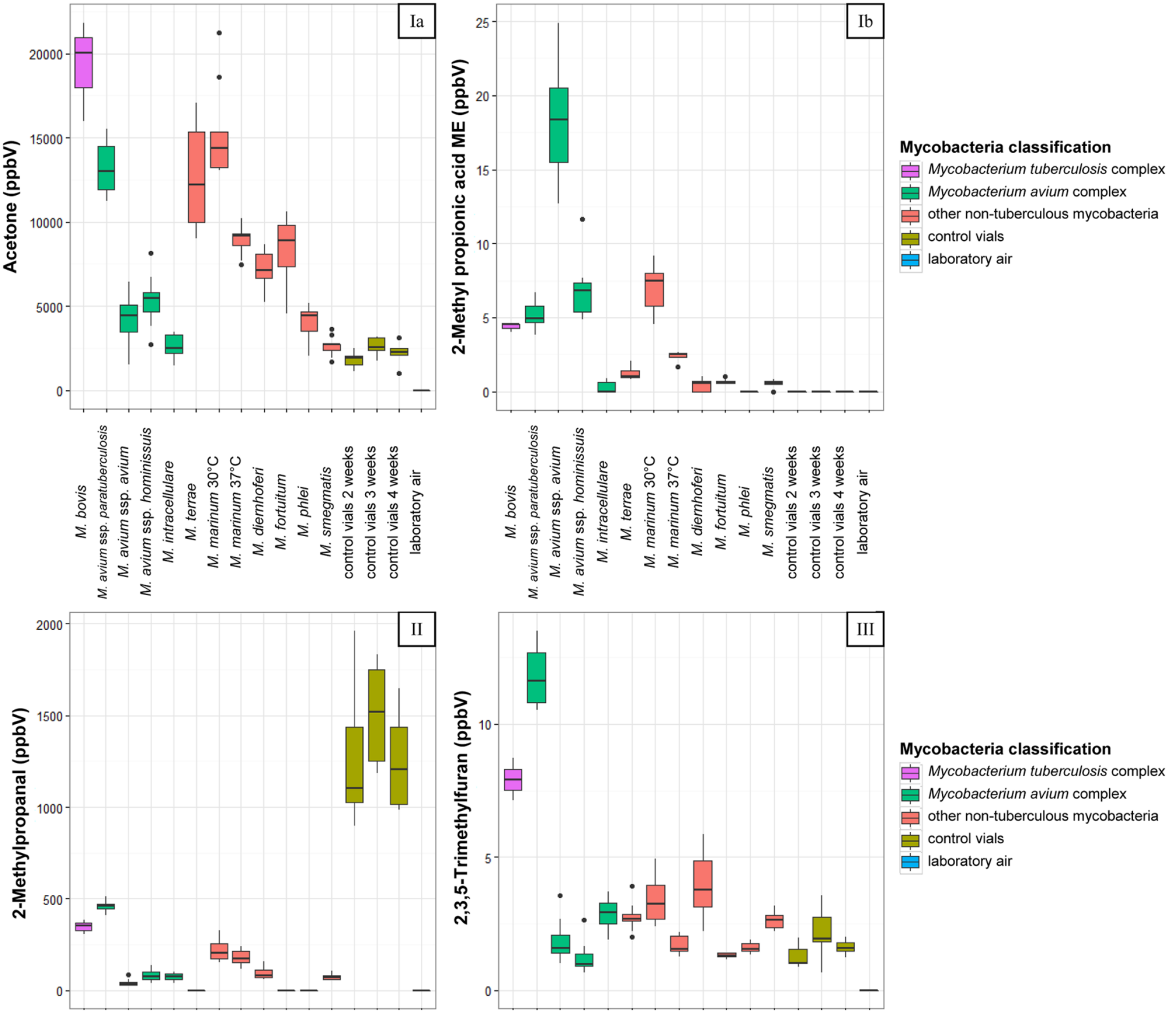
VOC concentrations above different mycobacterial species and pure media control slants inoculated with MB-Bouillon, forming four groups of substances. Ia—VOC concentrations above inoculated slants were higher than or equal to control vials; Ib—VOCs were detectable above inoculated slants only and not above control vials; II—VOC concentrations above inoculated slants were equal to or lower than above control slants; III—VOC concentrations above inoculated slants were higher than, equal to or lower than above control slants; ME–methyl ester.

VOCs were organised into four groups, stated in S3 Table and visualised in Fig 3:

(Ia) n = 13 concentrations of VOCs above inoculated slants were either higher or equal than above control vials

(Ib) n = 16 VOCs were only detectable above inoculated slants not above control vials

(II) n = 12 concentration of VOCs was higher or equally concentrated above control slants than above inoculated ones

(III) n = 13 VOCs with a concentration being higher, equal or lower above inoculated slants than above control slants

All eleven mycobacterial species could be differentiated from control vials by at least 21 VOCs (Table 2).

**Table 2:** VOC concentration above inoculated slants compared to control vials.

chemical class	volatile organic compound	MB	MAP	MAA	MAH	MI	MT	MM37	MM30	MD	MF	MP	MS
**Alcohols**	Ethanol	n.s.	n.s.	n.s.	n.s.	n.s.	n.s.	n.s.	n.s.	↑	n.s.	n.s.	n.s.
	2-Methylpropanol	↑	↑	n.s.	↑	↑	n.s.	↑	↑	↑	n.s.	n.s.	↑
	3-Methyl-1-butanol	n.s.	n.s.	n.s.	n.s.	↑	n.s.	↑	↑	↑	n.s.	n.s.	↑
	2-Methyl-1-butanol	n.s.	n.s.	n.s.	n.s.	↑	n.s.	n.s.	↑	↑	n.s.	n.s.	↑
	Phenylethylalcohol	n.s.	n.s.	n.s.	n.s.	↑	n.s.	↑	↑	↑	n.s.	n.s.	↑
	2-Propen-1-ol	n.s.	n.s.	n.s.	n.s.	↑	n.s.	n.s.	n.s.	↑	n.s.	n.s.	↑
	4-Methyl-1-pentanol	n.s.	n.s.	n.s.	n.s.	↑	n.s.	↑	↑	↑	n.s.	n.s.	↑
	3-Methyl-1-hexanol	n.s.	n.s.	n.s.	n.s.	↑	n.s.	n.s.	n.s.	↑	n.s.	n.s.	↑
	Pentanol	↓Ø	↓Ø	↓Ø	↓Ø	n.s.	↓	↓	↓	n.s.	↓	↓Ø	n.s.
	Hexanol	n.s.	n.s.	n.s.	n.s.	↑	n.s.	↑	n.s.	↑	n.s.	n.s.	↑
	2-Heptanol	↑	↑	n.s.	n.s.	n.s.	n.s.	n.s.	n.s.	↑	n.s.	n.s.	↑
	3-Octanol	↑	**Χ**	n.s.	n.s.	↑	n.s.	n.s.	n.s.	↑	n.s.	↑	↑
**Aldehydes**	Acetaldehyde	↓	↓	↓	↓	↓	↓	↓	↓	↓	↓	↓	↓
	2-Methylpropanal	↓	↓	↓	↓	↓	↓Ø	↓	↓	↓	↓Ø	↓Ø	↓
	3-Methylbutanal	↓	↓	↓	↓	↓	↓Ø	↓	↓	↓	↓Ø	↓Ø	↓
	2-Methylbutanal	↓	↓	↓	↓	↓	↓	↓	↓	↓	↓	↓Ø	↓
	Benzaldehyde	↓Ø	↓Ø	↓Ø	↓Ø	↓Ø	↓Ø	↓Ø	↓Ø	↓Ø	↓Ø	↓Ø	↓Ø
	Propanal	↓Ø	↓Ø	↓Ø	↓Ø	↓Ø	↓Ø	↓Ø	↓Ø	↓Ø	↓Ø	↓Ø	↓Ø
	Pentanal	↓Ø	↓Ø	↓Ø	↓Ø	↓Ø	↓Ø	↓Ø	↓Ø	↓Ø	↓Ø	↓Ø	↓Ø
	Hexanal	↓Ø	↓Ø	↓Ø	↓Ø	↓Ø	↓Ø	↓Ø	↓Ø	↓Ø	↓Ø	↓Ø	↓Ø
	Heptanal	↓Ø	↓Ø	↓Ø	↓Ø	↓Ø	↓Ø	↓Ø	↓Ø	↓Ø	↓Ø	↓Ø	↓Ø
**Hydro-carbons**	2,2-Dimethylbutane	n.s.	↑	n.s.	n.s.	n.s.	n.s.	n.s.	n.s.	n.s.	n.s.	n.s.	n.s.
	2,3-Dimethylbutane	n.s.	n.s.	n.s.	n.s.	n.s.	n.s.	n.s.	n.s.	n.s.	n.s.	n.s.	n.s.
	2-Methylpentane	n.s.	n.s.	n.s.	n.s.	n.s.	n.s.	n.s.	n.s.	↑	n.s.	n.s.	n.s.
	3-Methylpentane	n.s.	↑	n.s.	n.s.	n.s.	n.s.	n.s.	n.s.	↑	n.s.	n.s.	n.s.
	Pentane	↑	↑	↑	↑	↑	↑	↑	↑	↑	↑	↑	↑
	Heptane	↑	↑	n.s.	n.s.	↑	↑	↑	↑	↑	↑	n.s.	↑
	Octane	↑	↑	↑	n.s.	↑	↑	↑	↑	↑	↑	↑	↑
	Nonane	↑	↑	n.s.	n.s.	↑	↑	n.s.	↑	↑	n.s.	n.s.	↑
	Methylcyclopentane	n.s.	↑	n.s.	n.s.	↑	↑	↑	n.s.	↑	n.s.	n.s.	↑
	Hexane	↑	↑	n.s.	n.s.	↑	↑	↑	↑	↑	n.s.	n.s.	↑
**Ester**	2-Methyl-propionic acid ME	↑	↑	**Χ**	↑	n.s.	n.s.	↑	↑	n.s.	n.s.	n.s.	n.s.
	3-Methyl-1-butanol acetate	n.s.	n.s.	n.s.	n.s.	↑	n.s.	↑	↑	↑	n.s.	n.s.	↑
	Benzoic acid ME	n.s.	n.s.	**Χ**	n.s.	n.s.	n.s.	n.s.	n.s.	n.s.	n.s.	n.s.	n.s.
**Furans**	Furan	n.s.	n.s.	↓	↓	n.s.	n.s.	n.s.	n.s.	n.s.	n.s.	n.s.	n.s.
	2-Methylfuran	↓	**Χ**	↓	↓	↓	↓	↓	↓	↓	↓	↓	↓
	2-Ethylfuran	↓	n.s.	↓	↓	↓	↓	↓	↓	↓	↓	↓	↓
	2-Propylfuran	↓	**Χ**	↓Ø	↓Ø	↓Ø	↓Ø	n.s.	↓Ø	n.s.	↓Ø	↓Ø	↓Ø
	2,3,5-Trimethylfuran	↑	**Χ**	n.s.	n.s.	↑	n.s.	↑	n.s.	↑	n.s.	n.s.	↑
	2n-Butylfuran	n.s.	**Χ**	↓Ø	↓Ø	↓Ø	↓Ø	↓	↓Ø	n.s.	↓Ø	↓Ø	↓Ø
	Dibromochloromethane	↓	↓	↓	↓	↓	↓	n.s.	n.s.	n.s.	↓	↓	n.s.
**Ketones**	Acetone	↑	↑	↑	↑	n.s.	↑	↑	↑	↑	↑	n.s.	n.s.
	2,3-Butadione	n.s.	↑	n.s.	n.s.	n.s.	↑	↑	n.s.	n.s.	↑	n.s.	n.s.
	2-Butanone	↑	↑	↑	n.s.	↑	↑	**Χ**	↑	↑	↑	n.s.	↑
	2-Pentanone	↑	↑	↑	n.s.	↑	↑	**Χ**	↑	↑	↑	n.s.	n.s.
	3-Pentanone	↑	**Χ**	↑	↑	↑	↑	↑	↑	↑	n.s.	↑	n.s.
	Methylisobutylketone	n.s.	n.s.	n.s.	n.s.	↑	n.s.	n.s.	n.s.	↑	n.s.	n.s.	n.s.
	2-Heptanone	↓	↓	↓	↓	n.s.	↓	↓	↓	n.s.	n.s.	n.s.	↓
	3-Octanone	↑	↑	↑	↑	↑	↑	↑	↑	↑	n.s.	↑	↑
**N-containing compounds**	Acetonitrile	n.s.	n.s.	n.s.	n.s.	n.s.	n.s.	↑	n.s.	↑	n.s.	n.s.	n.s.
	2-Methylpropanenitrile	n.s.	n.s.	n.s.	n.s.	n.s.	n.s.	n.s.	n.s.	↑	n.s.	n.s.	↑
	2-Methylbutanenitrile	n.s.	n.s.	n.s.	n.s.	↓Ø	↑	↓Ø	↓Ø	↓Ø	↑	n.s.	↓Ø
	3-Methylbutanitrile	n.s.	n.s.	n.s.	n.s.	↑	↑	↑	↑	↑	n.s.	n.s.	↑
	Dimethyldisulfid	n.s.	n.s.	↓	↓	n.s.	↓	↓	↓	↑	↓Ø	↑	n.s.
	total count of VOCs	30	34	27	24	38	31	37	35	43	24	21	37

MAP—M. avium ssp. paratuberculosis, MAA—M. avium ssp. avium, MAH—M. avium ssp. hominissuis, MI—M. intracellulare, MT—M. terrae, MM37 –M. marinum (37°C), MM30 –M. marinum (30°C), MD—M. diernhoferi, MF—M. fortuitum, MP—M. phlei, MS—M. smegmatis, ME—methyl ester, **Χ** –‘Indicator substance’ each value of one species is higher than all values of all other species, ↑ –substance concentration above bacteria significant higher than above control slants, ↓ - substance concentration above bacteria significant lower than above control slants, Ø –substance concentration below level of detection, n.s.–not significant.

### Core-profile of Mycobacteriaceae

Seventeen substances showed the same tendencies for all mycobacteria. While the inoculated vials had eight volatiles (2-methylpropanol, 2-methyl-1-butanol, pentane, heptane, octane, 2,3-butadione, 3-pentanone, and 3-octanone) that presented higher concentrations than the control slants, they had nine VOCs (acetaldehyde, propanal, 3-methylbutanal, 2-methylbutanal, pentanal, hexanal, heptanal, benzaldehyde, and 2-methylpropanal) that decreased during the incubation period (see Fig 3II).

### Species-specific VOC profiles

‘Indicator substances’ are characterised by the fact that the concentration value of each individual measurement of a certain VOC above one species were higher than all individual values of all other species. Consequentially, the median concentration of this VOC above this species was significantly higher than median values of the other species (Table 2). MAP posed six indicator substances with the highest concentrations (for example 2,3,5-trimethylfuran (Fig 3III)), while for MAA the indicator substances were 2-methyl propionic acid ME (Fig 3Ib) and benzoic acid ME. Two VOCs were detectable as indicator substances above MM37.

Not only the highest concentration of a substance, but also specific concentration levels of substances indicate the presence of a particular species. For example the median concentration of 2,3,5-trimethylfuran (Fig 3III) was five times higher above MB, but seven times higher above MAP, each compared to the concentration above control vials. Another example was the concentration of 2-butanone, which was three times higher above MM30 and six times higher above MM37 compared to control slants (S3 Table).

All species of mycobacteria could be differentiated from each other by the concentrations of a certain number of VOCs, even though they formed clusters in the PCA (Fig 1). Concerning MI, MD, and MS, the concentrations of nine volatiles above MI were significantly lower than above MD. These were ethanol, 4-methyl-1-pentanol, furan, 2,3,5-trimethylfuran (Fig 3III), acetone (Fig 3Ia), acetonitrile, hexanol, 3-methyl-1-hexanol, and 3-methyl-1-butanol acetate. The concentrations of 2-methyl-1-butanol and octane above MI were significantly higher than above MD and MS. Also, 2-propen-1-ol, 2-methylpropanol, 3-methyl-1-butanol, heptane, 3-octanone, and methylisobutylketone were significantly more concentrated above MI than above MS. For the cluster of MAA, MAH, MP, and MF, three VOCs (2-butanone, 2-pentanone, and 3-pentanone) were significantly more concentrated above MAA than above MP. The substance 2-butanone was significantly higher concentrated above MAA than above MAH, while 2-methyl propionic acid ME (Fig 3Ib), benzoic acid ME—being the indicator substances for MAA -, and 3-pentanone were significantly less concentrated above all three species (MF, MP, and MAH). For these four species (MAA, MAH, MP, and MF), 2-methylpropanol was highest concentrated above MAH.

### Homogeneity within species

**Fig 4:**
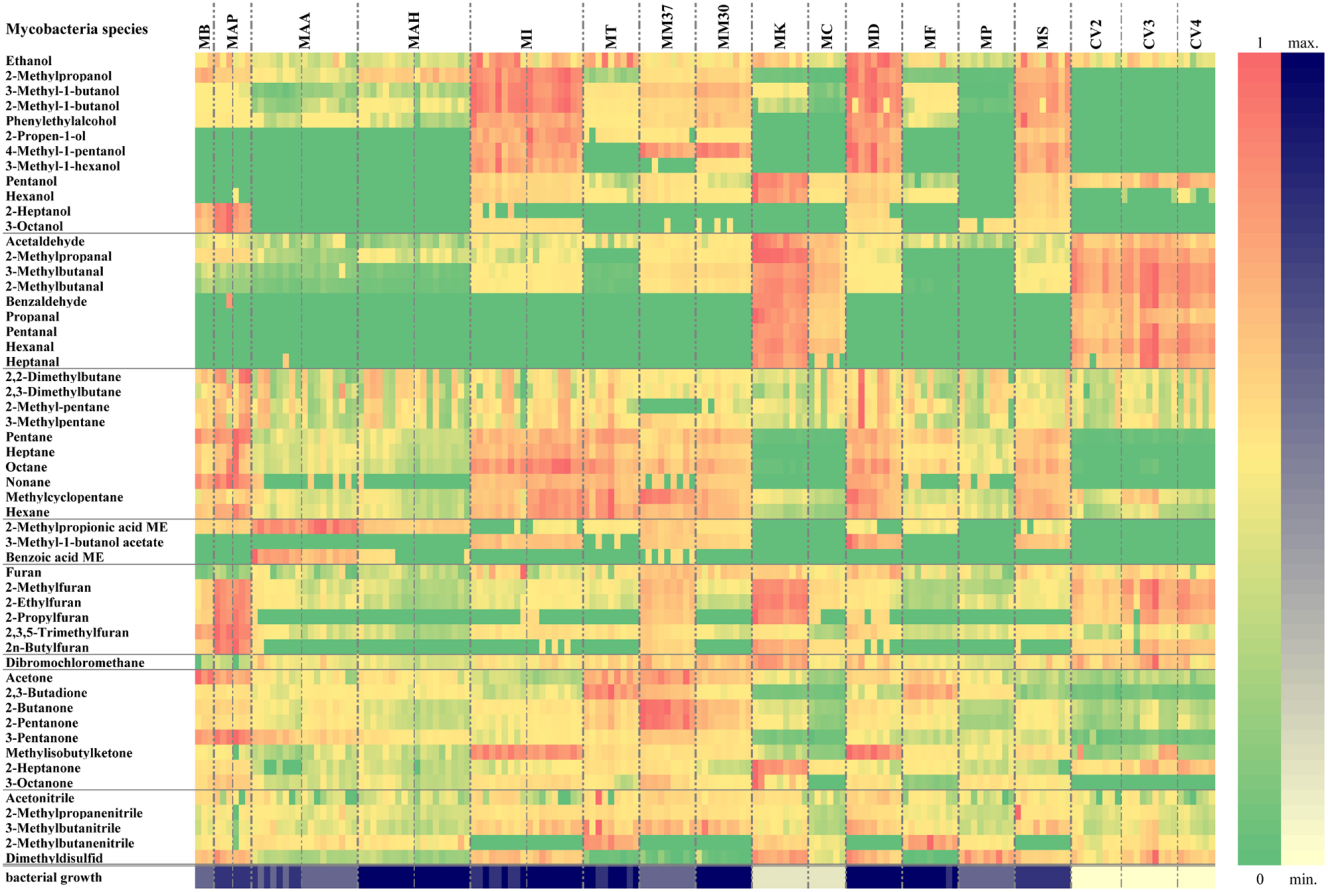
VOC emissions from different mycobacteria. The illustration is a heatmap with normalised data to a maximum of each substance. MAP–*M*. *avium* ssp. *paratuberculosis*; MAA–*M*. *avium* ssp. *avium*; MAH–*M*. *avium* ssp. *hominissuis*; MI–*M*. *intracellulare*; MT–*M*. *terrae*; MM37 –*M*. *marinum* (37°C); MM30 –*M*. *marinum* (30°C); MD–*M*. *diernhoferi*; MF–*M*. *fortuitum*; MP–*M*. *phlei*; MS–*M*. *smegmatis*; L2 –control vials incubated for 2 weeks; L3 –control vials incubated for 3 weeks; L4 –control vials incubated for 4 weeks; ME–methyl ester.

The strains of each species did not show significant differences in the concentration of most volatiles (Table 3). For MAP, VOCs did not differ significantly from each other. In the case of MAA it was 18 of 27, for MAH 18 of 24, and for MI 24 of 38. Fig 4 shows that the difference in VOC concentration between two strains of the same species was lower than among different species.

**Table 3:** Inter-strain variability of VOCs tested per species by means of Mann-Whitney-U-Test.

	p-value					p-value			
VOC	MAP	MAA	MAH	MI	VOC	MAP	MAA	MAH	MI
**2-Methylpropanol**	n.s.		n.s.	n.s.	**2,2-Dimethylbutane**	n.s.			
**3-Methyl-1-butanol**				n.s.	**3-Methylpentane**	n.s.			
**2-Methyl-1-butanol**				n.s.	**Pentane**	n.s.	n.s.	<0.05	n.s.
**Phenylethylalcohol**				n.s.	**Heptane**	n.s.			<0.01
**2-Propen-1-ol**				<0.001	**Octane**	n.s.	<0.05		<0.05
**4-Methyl-1-pentanol**				n.s.	**Nonane**	n.s.			<0.001
**3-Methyl-1-hexanol**				n.s.	**Methylcyclopentane**	n.s.			<0.05
**Pentanol**	n.s.	n.s.	n.s.	n.s.	**Hexane**	n.s.			<0.05
**2-Heptanol**	n.s.				**2-Methyl-propionic acid ME**	n.s.	n.s.	n.s.	
**3-Octanol**	n.s.			n.s.	**3-Methyl-1-butanol acetate**				n.s.
**Furan**		n.s.	n.s.		**Benzoic acid ME**		<0.001		
**2-Methylfuran**	n.s.	<0.01	<0.05	n.s.	**Dibromochloromethane**	n.s.	n.s.	n.s.	n.s.
**2-Ethylfuran**		<0.05	n.s.	n.s.	**Acetaldehyde**	n.s.	n.s.	n.s.	<0.05
**2-Propylfuran**	n.s.	n.s.	n.s.	n.s.	**2-Methylpropanal**	n.s.	<0.001	n.s.	n.s.
**2,3,5-Trimethylfuran**	n.s.			<0.05	**3-Methylbutanal**	n.s.	n.s.	<0.05	<0.01
**2n-Butylfuran**	n.s.	n.s.	n.s.	n.s.	**2-Methylbutanal**	n.s.	n.s.	n.s.	<0.01
**Acetone**	n.s.	n.s.	n.s.		**Benzaldehyde**	n.s.	n.s.	n.s.	n.s.
**2,3-Butadione**	n.s.				**Propanal**	n.s.	n.s.	n.s.	n.s.
**2-Butanone**	n.s.	<0.05		n.s.	**Pentanal**	n.s.	n.s.	n.s.	n.s.
**2-Pentanone**	n.s.	<0.01		<0.01	**Hexanal**	n.s.	n.s.	n.s.	n.s.
**3-Pentanone**	n.s.	<0.05	n.s.	<0.01	**Heptanal**	n.s.	n.s.	n.s.	n.s.
**Methylisobutylketone**				n.s.	**3-Methylbutanenitrile**				<0.05
**2-Heptanone**	n.s.	<0.01	<0.05		**2-Methylbutanenitrile**				n.s.
**3-Octanone**	n.s.	n.s.	<0.01	<0.01	**Dimethyldisulfid**		n.s.	<0.001	

Only substances, which were included in the species-specific VOC profile, were examined. MAP—M. avium ssp. paratuberculosis, MAA—M. avium ssp. avium, MAH—M. avium ssp. hominissuis, MI—M. intracellulare, n.s.–not significant, ME–methyl ester.

**Fig 5:**
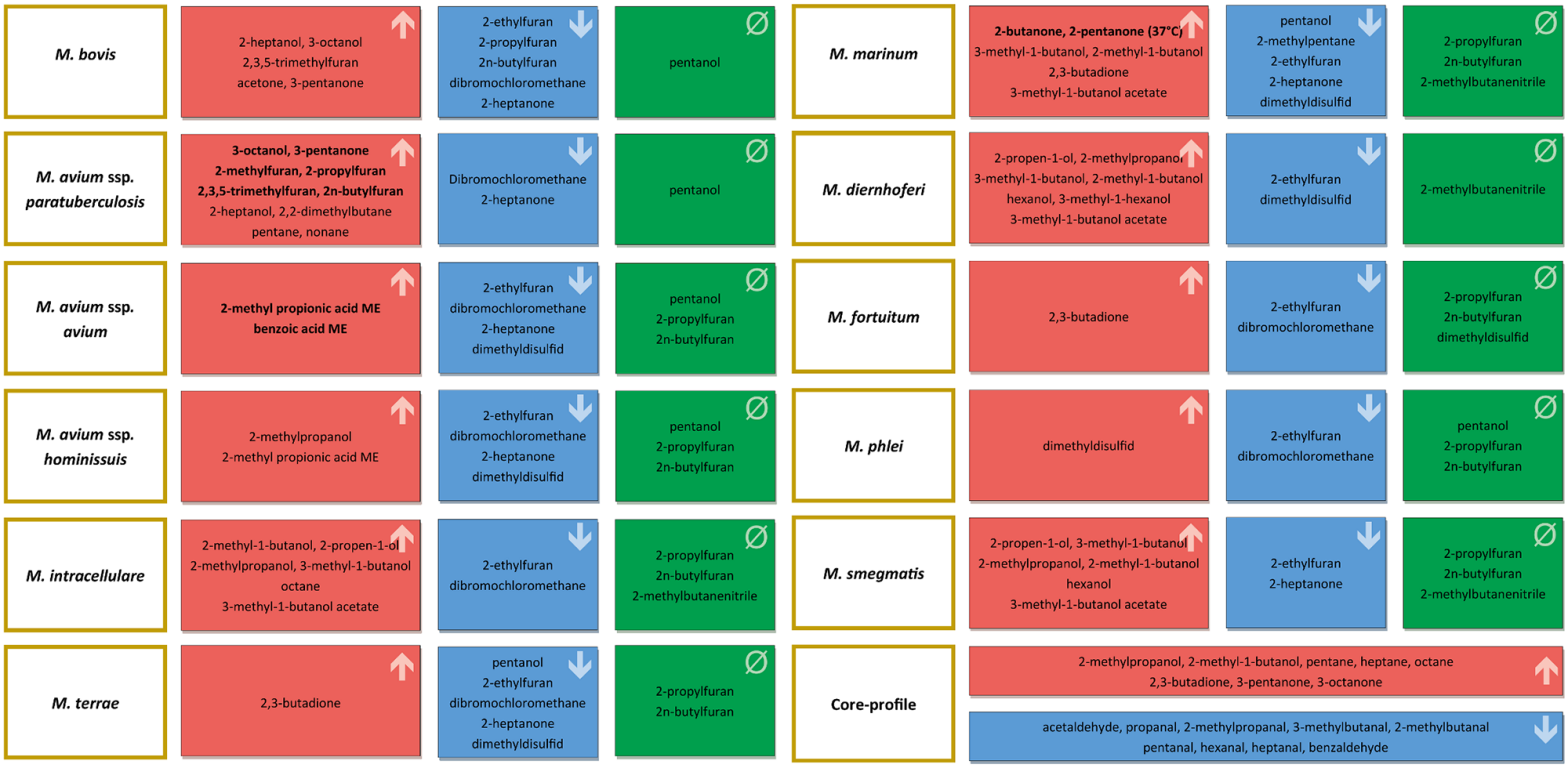
Suggested VOC profile consisting of the most influencing substances for each mycobacterial species. ↑ - substance is significantly higher above bacteria than above control vials; ↓ - substance is significantly lower above bacteria than above control vials; **Ø** –substance is not measurable above bacteria; bold: the values of the indicator substance of a species are higher than all values of all other species; ME–methyl ester.

Based on the results we propose following VOC profiles for each species (Fig 5).

## Discussion

As expected, data support the possibility of distinguishing inoculated slants from pure media control slants by means of VOC analysis, especially for MB, MAP, MAA, MAH, MI, MT, MM, MD, MF, MP, and MS.

### Identification of species

The results of the current study suggest that by taking indicator substances into account conclusions about the presence of corresponding *Mycobacterium* species can be drawn. Three of the species included in the present study produced at least two of these substances. Combining this with the concentration levels of other VOCs and the complete absence of some other volatiles, unequivocal VOC profiles can be defined for all of the included species (Fig 5). Keeping in mind that these profiles have been defined with only a small selection of specific mycobacteria and measured at specific stages of bacterial growth, further investigations have to address the VOC profiles of these species and other species using different lengths and conditions of incubation.

For MAP, findings of the current study support previously published results [48] where we found 31 VOCs with significant differences compared to control slants after 4 weeks of incubation. In the current study, 34 VOCs had increased or decreased significantly after 4 weeks of incubation. Twenty-one volatiles of the MAP profile defined in the previous study were confirmed in the current study. This time we included 13 additional substances in the MAP-specific VOC profile, which had not been detected before. On the other hand, there were 10 VOCs included in the MAP-specific VOC profile in the previous study, which were excluded from the current study because of the high variability in their concentrations above control vials (see section 2.4 Selection of VOCs). Due to different study designs and the number of comparative groups, different statistical tests had to be used and could, therefore, explain the differences of the defined MAP-specific VOC profile. Based on the study of Trefz et al. [38], the importance of furans in a MAP-specific VOC profile was assumed. In the current study, the concentrations of four furans were not only significantly higher above MAP than above control, but every sample of MAP inoculated vials showed higher concentrations than all the other samples (see Table 2, ‘Indicator substances’).

Pentanal was found to be significantly different for each species in the study of Mellors et al. [45], that is *M*. *avium* (subspecies not designated), *M*. *bovis* BCG, MI, and *M*. *xenopi*. Our results show a significant decrease in pentanal concentration above the inoculated slants of all the species, see 3.2 Core-profile. A study by Nawrath et al. [53] incorporated different species of mycobacteria including, MS and MAA amongst others. Compared to their results, only one substance was also found significant in our study for MS: i.e. hexanol. A possible explanation for the different outcomes of the studies is that various incubation protocols were used. Not only do the media and media types (solid compared to liquid) differ from our protocols, but so does the length of incubation. In addition, different methods for pre-concentration and analysis of volatiles have been used. Nawrath et al. [53] used closed-loop stripping analysis combined with GC-MS, while Mellors et al. [45] used solid-phase micro-extraction and analysed via two-dimensional gas chromatography time-of-flight mass spectrometry. Due to different packing materials in the devices used for micro-extraction, the VOCs from different chemical classes bind the trapping devices in variable quality [54].

In a previous study we assessed different culture conditions including bacterial density, duration of incubation, and media type [48]. Here another methodological factor, i.e. incubation temperature, was addressed: MM30 and MM37 were incubated at different temperatures. The VOC patterns above those slants differed significantly even though it was the same species (Figs 1 and 4).

### Classification of mycobacteria

The scattering in the PCA (Fig 1) does not reflect the most common classification scheme for mycobacteria. In this scheme developed by Ernest H. Runyon [12], mycobacteria are grouped by their phenotype. This includes pigment production that is dependent on light and bacterial growth rate. Pigments include photochromogens (group I, MK, MM), scotochromogens (group II), and non-chromogens (groups III & IV). Group III contains slow growing mycobacteria (MAA, MAH, MAP, MI, MB) and group IV rapid growing mycobacteria (MT, MC, MD, MF, MP, MS). In addition, there are ungrouped mycobacteria. The overlays also do not correspond to the conventional classification separating MTC, MAC, and other NTMs [55]. The clustering in the PCA corresponds more to the groups classified by lipid composition published by Lechevalier et al. [56]. For example, after the pyrolysis of the mycolic esters of the cell wall of MD and MS in the gas chromatograph, a mixture of mainly unbranched saturated fatty esters with 22 and 24 carbons are released. Other mycobacterial species show a different composition of lipids. Studies addressing the patterns of mycolic acids in the cell wall of mycobacteria suggest using these acids for bacterial classification and identification [57,58]. These studies present the same pattern consisting of alpha, keto mycolic acids, and wax esters for MAA, MAH, and MP, which belong to the same PCA cluster in our study. On the other hand, MI consists of the same mycolic acid pattern, but presents a different result in our PCA. Once again, the impact of culture conditions such as bacterial density, length of incubation, and the stage of bacterial growth are considerable. Since the species were propagated, inoculated, and sampled at different days, methodological factors can be neglected as reason for the clustering in the PCA.

### Value for a diagnostic approach

Significant differences among the strains of the same species presented in Table 3 can be partly explained by the different stages of bacterial growth at the time point of analysis, especially for MAA and MI (see Fig 4). A few studies have addressed the kinetics of the volatile profile during different stages of bacterial growth, and have shown that the substance concentrations increase and/or decrease over time [48,49].

Due to poor bacterial growth of MK and MC at the time of VOC analysis, probably because HEYM is not the best-adapted medium for these species, measurements needed to be excluded from further statistical analysis. Even though they showed significant differences compared to control vials, the inclusion criterion required that they exhibit a growth intensity of at least 1 point and 3 points for ideal comparison. Our results confirm an earlier study [48] that volatile emissions from bacteria are measurable long before growth is visually apparent. In both studies, the aldehyde concentrations increased significantly before it decreased, at the time when the cultures first became visible, until the aldehydes above the bacteria were no longer measurable, as compared to control vials (see Fig 4).

Defining a set of volatile substances resembling the presence of any species of mycobacteria could be a helpful tool for future diagnostic application. Other studies have approached this issue as well (e.g. for *E*. *coli*) [59]. Further investigations are necessary to discriminate these 17 VOCs from substances that indicate the presence of growing mycobacteria in general or display species of the same suborder, for example *Corynebacterineae*.

## Conclusions

This study revealed species-specific VOC profiles for eleven mycobacterial species that showed visually apparent bacterial growth at the time point of analysis. We were able to distinguish VOCs above inoculated vials compared to pure media control slants and VOCs that showed differing patterns above the different *Mycobacterium* species. Therefore, compared to control vials, a core-profile of Mycobacteriaceae could be defined that contains eight increasing and nine decreasing substances. The presence of all 13 species could be proven by means of VOC analysis.

From a diagnostic perspective, inter-strain variability is negligible. VOC emissions seem to correspond strongly with the cell wall structure and particularly the lipid composition of the cell wall. Nevertheless, in comparison to previous studies and the literature, culture conditions and methodological factors seem to have a great impact. This is important information for future developments towards *in vitro* testing of bacterial growth in general. The results indicate that analysis of volatile organic compounds could accelerate and simplify diagnostic methods for Mycobacteriaceae.

How conclusive the results are for other mycobacterial species *in vitro* and for infections *in vivo*, respectively, remains to be elucidated in future research.

## Supporting information

S1 Table Manufacturer of reference substances. (DOCX)

S2 Table Reference substances used for identification and quantification of selected VOCs. VOC–volatile organic compound; R2 –coefficient of determination; LOD–limit of detection; LOQ–limit of quantification; ppbV–parts per billion by volume.
(DOCX)

S3 Table Median and percentiles (0.25 and 0.75) of the concentration of volatile organic compounds in ppbV and Tukey-HSD-test. (PDF)
