# Supplementary material for: Comparative analysis of volatile organic compounds for the classification and identification of mycobacterial species
Source: PLoS One. 2018 Mar 20;13(3):e0194348. doi: 10.1371/journal.pone.0194348 (PMC5860768; doi:10.1371/journal.pone.0194348)
Supplement: S1 Table — (DOCX) [file pone.0194348.s001.docx]

| **VOCs** | **Manufacturer** | **Head Office** |
| --- | --- | --- |
| 1-Hexanol | Fluka/Sigma-Aldrich | Steinheim, Germany |
| 2,2-Dimethylbutane |  |  |
| 2,3-Butanedione |  |  |
| 2,3-Dimethylbutane |  |  |
| 2-Ehtylfuran |  |  |
| 2-Heptanone |  |  |
| 2-Methyl-1-Butanol |  |  |
| 2-Methyl-Butanenitrile |  |  |
| 2-Methylfuran |  |  |
| 2-Methylpentane |  |  |
| 2-Methylpropanal |  |  |
| 2-Methylpropanol |  |  |
| 2-Pentanone |  |  |
| 2-Propen-1-ol |  |  |
| 3-Methyl-1-Butanol |  |  |
| 3-Methyl-1-butanol acetate |  |  |
| 3-Methylbutanenitrile |  |  |
| 3-Octanol |  |  |
| 3-Octanone |  |  |
| 4-Methylpentanol |  |  |
| Benzaldehyde |  |  |
| Dibromochloromethane |  |  |
| Ethanol |  |  |
| Furan |  |  |
| Heptanal |  |  |
| Heptane |  |  |
| Methylcyclopentane |  |  |
| Methylisobutylketone |  |  |
| Phenylethylalcohol |  |  |
| Propanal |  |  |
| 2-Methylbutanal | Abbott GmbH and CoKG | Wiesbaden, Germany |
| Acetaldehyde |  |  |
| Dimethyldisulfid |  |  |
| 2-Propylfuran | TCI Europe N.V. | Zwijndrecht, Belgium |
| 2-Heptanol |  |  |
| 2-methylpropanenitrile |  |  |
| 2n-Butylfuran |  |  |
| 3-Methylbutanal |  |  |
| 3-Methylhexanol |  |  |
| 3-Methylpentane |  |  |
| Pentanol |  |  |
| 2-Butanone | Ionimed Analytik GmbH | Innsbruck, Austria |
| Acetone |  |  |
| Hexanal |  |  |
| 3-Pentanone | Supelco | Bellefonte, USA |
| Hexane |  |  |
| Pentane |  |  |
| Nonane | Merck KGaA | Darmstadt, Germany |
| Octane |  |  |
| 2,3,5-Trimethylfuran | Georganics Ltd. | Pezinok, Slovak republic |
| 2-Methyl-propanoic acid methyl ester | Bruckner Analysetechnik | Linz, Austria |
| Benzioc acid methyl ester | Agilent | Santa Clara, USA |
| Acetonitrile | VWR Prolabo Chemicals | Radnor, USA |
