# Supplementary material for: Comparative analysis of volatile organic compounds for the classification and identification of mycobacterial species
Source: PLoS One. 2018 Mar 20;13(3):e0194348. doi: 10.1371/journal.pone.0194348 (PMC5860768; doi:10.1371/journal.pone.0194348)
Supplement: S2 Table — VOC–volatile organic compound; R2 –coefficient of determination; LOD–limit of detection; LOQ–limit of quantification; ppbV–parts per billion by volume. (DOCX) [file pone.0194348.s002.docx]

| **Chemical class** | **VOC** | **retention time (min)** | **R²** | **LOD (ppbV)** | **LOQ (ppbV)** |
| --- | --- | --- | --- | --- | --- |
| Alcohol | Ethanol | 6.64 | 0.97 | 26.00 | 50.82 |
|  | 2-Propen-1-ol | 9.72 | 0.98 | 0.08 | 0.18 |
|  | 2-Methylpropanol | 12.11 | 0.99 | 0.54 | 1.16 |
|  | 3-Methyl-1-butanol | 15.45 | 0.99 | 0.68 | 1.38 |
|  | 2-Methyl-1-butanol | 15.66 | 0.99 | 0.26 | 0.57 |
|  | Pentanol | 16.44 | 0.99 | 1.00 | 2.18 |
|  | 4-Methyl-1-pentanol | 18.69 | 0.98 | 0.46 | 1.03 |
|  | Hexanol | 19.46 | 1.00 | 0.42 | 0.83 |
|  | 2-Heptanol | 20.37 | 1.00 | 0.69 | 1.25 |
|  | 3-Methyl-1-hexanol | 21.42 | 1.00 | 0.92 | 1.75 |
|  | 3-Octanol | 22.57 | 0.98 | 0.85 | 1.66 |
|  | Phenylethylalcohol | 26.27 | 0.97 | 3.34 | 6.10 |
| Aldehyde | Acetaldehyde | 4.70 | 0.98 | 47.35 | 159.27 |
|  | Benzaldehyde | 22.77 | 1.00 | 1.61 | 3.36 |
|  | Propanal | 7.24 | 0.95 | 12.08 | 24.97 |
|  | 2-Methylpropanal | 9.23 | 1.00 | 2.03 | 3.84 |
|  | 3-Methylbutanal | 12.60 | 0.98 | 0.27 | 0.48 |
|  | 2-Methylbutanal | 12.85 | 1.00 | 1.83 | 3.66 |
|  | Pentanal | 13.98 | 0.99 | 2.84 | 5.10 |
|  | Hexanal | 17.33 | 0.95 | 9.86 | 22.75 |
|  | Heptanal | 20.46 | 1.00 | 1.78 | 3.51 |
| Alkane | Pentane | 6.06 | 0.96 | 5.17 | 12.04 |
|  | Heptane | 12.53 | 1.00 | 0.11 | 0.25 |
|  | Octane | 15.68 | 1.00 | 0.24 | 0.56 |
|  | Nonane | 18.95 | 1.00 | 0.11 | 0.21 |
|  | 2,2-Dimethylbutane | 7.04 | 0.99 | 1.06 | 2.42 |
|  | 2,3-dimethylbutane | 7.98 | 1.00 | 0.07 | 0.15 |
|  | 2-Methylpentane | 8.07 | 1.00 | 1.15 | 2.71 |
|  | 3-Methylpentane | 8.58 | 0.98 | 0.44 | 0.96 |
|  | Methylcyclopentane | 10.33 | 1.00 | 0.06 | 0.13 |
|  | Hexane | 9.12 | 0.99 | 0.06 | 0.13 |
| Ester | 2-Methyl-propionic acid ME | 13.10 | 1.00 | 0.24 | 0.44 |
|  | 3-Methyl-1-butanol acetate | 19.21 | 1.00 | 0.11 | 0.24 |
|  | Benzoic acid ME | 25.34 | 1.00 | 0.61 | 1.29 |
| Furan | Furan | 6.71 | 0.95 | 3.82 | 8.08 |
|  | 2-Methylfuran | 10.07 | 0.99 | 0.39 | 0.87 |
|  | 2-Ethylfuran | 13.31 | 1.00 | 0.18 | 0.39 |
|  | 2-Propylfuran | 16.13 | 1.00 | 0.51 | 0.81 |
|  | 2,3,5-Trimethylfuran | 19.90 | 1.00 | 0.10 | 0.21 |
|  | 2n-Butylfuran | 19.43 | 1.00 | 0.18 | 0.38 |
| Halogenated Comp. | Dibromochloromethane | 17.45 | 1.00 | 0.06 | 0.15 |
| Ketone | Acetone | 7.44 | 0.99 | 15.87 | 29.11 |
|  | 2,3-Butadione | 10.57 | 1.00 | 1.70 | 3.44 |
|  | 2-Butanone | 10.83 | 1.00 | 1.55 | 3.13 |
|  | 2-Pentanone | 13.80 | 1.00 | 0.53 | 1.13 |
|  | 3-Pentanone | 14.03 | 1.00 | 0.36 | 0.78 |
|  | Methylisobutylketone | 15.40 | 0.98 | 0.07 | 0.12 |
|  | 2-Heptanone | 20.23 | 0.98 | 0.30 | 0.65 |
|  | 3-Octanone | 22.60 | 1.00 | 0.44 | 0.89 |
| Nitrogen containing compounds | Acetonitrile | 8.18 | 1.00 | 8.39 | 16.44 |
|  | 2-Methylpropanenitrile | 12.44 | 0.99 | 0.16 | 0.33 |
|  | 2-Methylbutanenitrile | 15.57 | 0.99 | 0.17 | 0.37 |
|  | 3-Methylbutanitrile | 15.90 | 0.99 | 0.13 | 0.28 |
| Sulfur containing comp. | Dimethyldisulfid | 15.30 | 0.75 | 0.15 | 0.29 |
|  | | | | | |
