# Supplementary material for: Comparative analysis of volatile organic compounds for the classification and identification of mycobacterial species
Source: PLoS One. 2018 Mar 20;13(3):e0194348. doi: 10.1371/journal.pone.0194348 (PMC5860768; doi:10.1371/journal.pone.0194348)
Supplement: S3 Table — (PDF) [file pone.0194348.s003.pdf]

median<br>(percentiles)<br>Tukey-HSD-test	*M. bovis*	*M. avium* ssp. *paratuberculosis*	*M. avium* ssp. *avium*	*M. avium* ssp. *hominissuis*	*M. intracellulare*	*M. terrae*	*M. marinum* incubated at 37°C	*M. marinum* incubated at 30°C	*M. diernhoferi*	*M. fortuitum*	*M. phlei*	*M. smegmatis*	control vials incubated for 2 weeks	control vials incubated for 3 weeks	control vials incubated for 4 weeks	laboratory air	group
**Ethanol**	371.234,87<br>(368,027.09 - 405,164.61)<br>bcde	399.112,89<br>(367,101.48 - 455,119.30)<br>bcd	292.528,34<br>(234,878.18 - 331,987.90)<br>e	288.207,92<br>(268,298.95 - 299,623.90)<br>e	458.866,37<br>(405,708.33 - 534,140.96)<br>bc	492.401,61<br>(441,861.99 - 505,687.89)<br>ab	387.258,80<br>(343,869.24 - 392,225.83)<br>cde	378.194,16<br>(354,033.58 - 391,767.96)<br>bcde	580.881,39<br>(530,926.36 - 595,082.03)<br>a	388.770,03<br>(320,471.76 - 397,360.46)<br>cde	267.336,20<br>(228,299.08 - 294,873.54)<br>e	448.334,79<br>(422,692.96 - 559,548.40)<br>abc	382.146,65<br>(315,189.62 - 401,686.33)<br>cde	383.862,97<br>(374,369.07 - 427,435.14)<br>bcd	305.957,70<br>(291,674.96 - 316,453.86)<br>de	0,00<br>(0.00 - 41.52)<br>f	III
**2-Propen-1-ol**	0,00<br>(0.00 - 0.00)<br>c	0,00<br>(0.00 - 0.00)<br>c	0,00<br>(0.00 - 0.00)<br>c	0,00<br>(0.00 - 0.00)<br>c	4,99<br>(3.95 - 5.45)<br>a	0,39<br>(0.34 - 0.60)<br>c	0,29<br>(0.27 - 0.39)<br>c	0,26<br>(0.24 - 0.34)<br>c	4,87<br>(4.81 - 5.81)<br>a	0,00<br>(0.00 - 0.00)<br>c	0,00<br>(0.00 - 0.00)<br>c	2,72<br>(2.16 - 2.87)<br>b	0,00<br>(0.00 - 0.00)<br>c	0,00<br>(0.00 - 0.00)<br>c	0,00<br>(0.00 - 0.00)<br>c	0,00<br>(0.00 - 0.00)<br>c	Ib
**2-Methylpropanol**	2.755,55<br>(2,672.71 - 2,956.31)<br>b	1.622,89<br>(1,504.32 - 1,660.84)<br>cd	517,44<br>(444.53 - 595.65)<br>ef	1.940,08<br>(1,712.04 - 2,015.68)<br>c	4.105,56<br>(3,819.43 - 4,568.96)<br>a	247,23<br>(222.86 - 278.11)<br>fg	991,58<br>(916.62 - 1,031.36)<br>de	1.407,23<br>(1,401.91 - 1,439.52)<br>cd	3.811,41<br>(3,590.87 - 5,092.41)<br>a	109,74<br>(97.34 - 122.00)<br>fg	12,36<br>(11.55 - 12.97)<br>fg	2.710,57<br>(2,585.59 - 3,216.87)<br>b	3,08<br>(2.42 - 3.71)<br>fg	3,69<br>(3.41 - 5.07)<br>fg	3,31<br>(3.02 - 4.17)<br>fg	0,00<br>(0.00 - 0.00)<br>g	Ia
**3-Methyl-1-butanol**	217,82<br>(188.40 - 236.83)<br>e	186,21<br>(132.48 - 203.04)<br>e	39,78<br>(21.86 - 48.43)<br>e	75,80<br>(61.14 - 92.67)<br>e	30.355,71<br>(24,212.21 - 32,438.13)<br>a	2.855,23<br>(2,451.00 - 2,942.48)<br>e	10.415,84<br>(9,113.56 - 10,780.43)<br>d	16.218,28<br>(15,426.30 - 17,785.31)<br>c	32.152,91<br>(28,570.25 - 33,591.87)<br>a	1.376,52<br>(1,265.29 - 1,629.61)<br>e	0,00<br>(0.00 - 0.00)<br>e	22.582,30<br>(19,269.87 - 23,688.01)<br>b	0,00<br>(0.00 - 0.00)<br>e	0,00<br>(0.00 - 0.00)<br>e	0,00<br>(0.00 - 0.00)<br>e	0,00<br>(0.00 - 0.00)<br>e	Ib
**2-Methyl-1-butanol**	145,05<br>(143.02 - 154.46)<br>d	86,77<br>(76.25 - 95.64)<br>d	25,47<br>(18.27 - 38.21)<br>d	54,56<br>(50.72 - 61.21)<br>d	11.722,24<br>(9,996.97 - 12,600.61)<br>a	600,86<br>(509.81 - 636.72)<br>d	1.790,48<br>(1,766.20 - 2,131.64)<br>cd	3.578,36<br>(3,539.51 - 4,273.04)<br>c	9.616,84<br>(8,651.44 - 12,083.47)<br>b	294,50<br>(272.31 - 325.07)<br>d	5,04<br>(3.82 - 5.84)<br>d	7.730,99<br>(6,898.00 - 9,285.74)<br>b	0,00<br>(0.00 - 0.00)<br>d	0,00<br>(0.00 - 0.00)<br>d	0,00<br>(0.00 - 0.00)<br>d	0,00<br>(0.00 - 0.00)<br>d	Ib
**Pentanol**	0,00<br>(0.00 - 0.00)<br>e	0,00<br>(0.00 - 0.00)<br>e	0,00<br>(0.00 - 0.00)<br>e	0,00<br>(0.00 - 0.00)<br>e	39,35<br>(31.51 - 41.23)<br>cd	5,55<br>(4.29 - 6.61)<br>e	22,64<br>(19.30 - 29.92)<br>d	6,99<br>(6.19 - 7.49)<br>e	43,23<br>(36.27 - 50.13)<br>c	4,67<br>(3.95 - 5.25)<br>e	0,00<br>(0.00 - 0.00)<br>e	42,73<br>(38.36 - 46.38)<br>c	33,98<br>(27.66 - 40.33)<br>cd	57,09<br>(50.42 - 90.53)<br>b	78,97<br>(73.81 - 109.97)<br>a	0,00<br>(0.00 - 0.00)<br>e	III
**4-Methyl-1-pentanol**	0,00<br>(0.00 - 0.00)<br>d	0,00<br>(0.00 - 0.00)<br>d	0,00<br>(0.00 - 0.00)<br>d	0,00<br>(0.00 - 0.00)<br>d	4,55<br>(4.03 - 5.23)<br>c	0,00<br>(0.00 - 0.00)<br>d	4,93<br>(4.10 - 6.14)<br>abc	6,92<br>(6.58 - 7.25)<br>a	5,11<br>(4.44 - 8.74)<br>ab	0,00<br>(0.00 - 0.00)<br>d	0,00<br>(0.00 - 0.00)<br>d	4,61<br>(3.78 - 5.73)<br>bc	0,00<br>(0.00 - 0.00)<br>d	0,00<br>(0.00 - 0.00)<br>d	0,00<br>(0.00 - 0.00)<br>d	0,00<br>(0.00 - 0.00)<br>d	Ib
**Hexanol**	0,00<br>(0.00 - 0.00)<br>d	0,00<br>(0.00 - 0.00)<br>d	0,00<br>(0.00 - 0.00)<br>d	0,00<br>(0.00 - 0.00)<br>d	39,17<br>(34.33 - 47.45)<br>b	4,30<br>(2.58 - 6.69)<br>d	14,74<br>(12.03 - 27.87)<br>c	7,95<br>(6.52 - 8.43)<br>d	51,82<br>(41.51 - 65.64)<br>a	3,94<br>(3.33 - 5.29)<br>d	0,00<br>(0.00 - 0.00)<br>d	39,74<br>(36.41 - 49.36)<br>b	0,00<br>(0.00 - 0.00)<br>d	0,00<br>(0.00 - 0.00)<br>d	1,63<br>(0.38 - 2.42)<br>d	0,00<br>(0.00 - 0.00)<br>d	Ib
**2-Heptanol**	5,60<br>(5.00 - 6.44)<br>b	9,80<br>(7.81 - 11.22)<br>a	0,00<br>(0.00 - 0.00)<br>d	0,00<br>(0.00 - 0.00)<br>d	0,00<br>(0.00 - 0.00)<br>d	0,00<br>(0.00 - 0.00)<br>d	0,00<br>(0.00 - 0.00)<br>d	0,00<br>(0.00 - 0.00)<br>d	2,01<br>(1.42 - 2.32)<br>c	0,00<br>(0.00 - 0.00)<br>d	0,00<br>(0.00 - 0.00)<br>d	1,97<br>(1.72 - 2.23)<br>c	0,00<br>(0.00 - 0.00)<br>d	0,00<br>(0.00 - 0.00)<br>d	0,00<br>(0.00 - 0.00)<br>d	0,00<br>(0.00 - 0.00)<br>d	Ib
**3-Methyl-1-hexanol**	0,00<br>(0.00 - 0.00)<br>c	0,00<br>(0.00 - 0.00)<br>c	0,00<br>(0.00 - 0.00)<br>c	0,00<br>(0.00 - 0.00)<br>c	48,89<br>(40.45 - 57.58)<br>b	0,00<br>(0.00 - 0.00)<br>c	0,00<br>(0.00 - 0.00)<br>c	6,30<br>(5.94 - 8.76)<br>c	71,90<br>(49.03 - 76.70)<br>a	0,00<br>(0.00 - 0.00)<br>c	0,00<br>(0.00 - 0.00)<br>c	43,65<br>(42.32 - 60.63)<br>b	0,00<br>(0.00 - 0.00)<br>c	0,00<br>(0.00 - 0.00)<br>c	0,00<br>(0.00 - 0.00)<br>c	0,00<br>(0.00 - 0.00)<br>c	Ib
**3-Octanol**	12,86<br>(11.19 - 13.73)<br>b	25,83<br>(19.29 - 30.56)<br>a	0,00<br>(0.00 - 0.00)<br>f	0,00<br>(0.00 - 0.00)<br>f	3,76<br>(3.36 - 4.14)<br>c	0,00<br>(0.00 - 0.00)<br>f	0,00<br>(0.00 - 0.00)<br>ef	0,00<br>(0.00 - 1.74)<br>def	4,21<br>(2.76 - 4.57)<br>c	0,00<br>(0.00 - 0.00)<br>f	3,16<br>(2.33 - 3.53)<br>cde	3,01<br>(2.63 - 4.22)<br>cd	0,00<br>(0.00 - 0.00)<br>f	0,00<br>(0.00 - 0.00)<br>f	0,00<br>(0.00 - 0.00)<br>f	0,00<br>(0.00 - 0.00)<br>f	Ib
**Phenylethyl-alcohol**	30,75<br>(30.02 - 36.98)<br>cd	26,44<br>(25.40 - 31.51)<br>cd	15,73<br>(11.06 - 17.40)<br>d	9,08<br>(7.23 - 11.74)<br>d	401,41<br>(355.59 - 427.29)<br>a	25,02<br>(21.21 - 29.38)<br>d	119,00<br>(103.81 - 123.86)<br>c	196,00<br>(181.21 - 260.55)<br>b	444,83<br>(424.89 - 510.48)<br>a	11,08<br>(10.06 - 14.44)<br>d	0,00<br>(0.00 - 0.00)<br>d	365,91<br>(329.63 - 376.73)<br>a	0,00<br>(0.00 - 0.00)<br>d	0,00<br>(0.00 - 0.00)<br>d	0,00<br>(0.00 - 0.00)<br>d	0,00<br>(0.00 - 0.00)<br>d	Ib
**Acetaldehyde**	1.590,83<br>(1,502.46 - 1,663.12)<br>cdef	1.744,69<br>(1,579.43 - 1,894.89)<br>cdef	1.066,41<br>(688.72 - 1,265.07)<br>def	847,71<br>(542.95 - 949.74)<br>ef	1.986,18<br>(1,512.59 - 2,697.91)<br>cd	1.842,22<br>(1,249.95 - 1,886.83)<br>cde	3.052,76<br>(2,765.36 - 3,505.75)<br>c	2.549,25<br>(2,426.01 - 2,833.56)<br>cd	2.615,15<br>(2,158.49 - 2,969.83)<br>cd	1.473,44<br>(1,126.91 - 1,972.96)<br>cdef	915,31<br>(738.80 - 1,251.42)<br>def	1.659,12<br>(1,420.81 - 2,336.50)<br>cde	6.570,90<br>(5,764.08 - 9,162.67)<br>b	10.167,66<br>(9,105.78 - 11,462.48)<br>a	7.256,22<br>(5,607.90 - 7,856.45)<br>b	0,00<br>(0.00 - 0.00)<br>f	II
**Propanal**	0,00<br>(0.00 - 0.00)<br>c	0,00<br>(0.00 - 0.00)<br>c	0,00<br>(0.00 - 0.00)<br>c	0,00<br>(0.00 - 0.00)<br>c	0,00<br>(0.00 - 0.00)<br>c	0,00<br>(0.00 - 0.00)<br>c	0,00<br>(0.00 - 0.00)<br>c	0,00<br>(0.00 - 0.00)<br>c	0,00<br>(0.00 - 0.00)<br>c	0,00<br>(0.00 - 0.00)<br>c	0,00<br>(0.00 - 0.00)<br>c	0,00<br>(0.00 - 0.00)<br>c	145,24<br>(135.56 - 204.26)<br>b	243,44<br>(193.36 - 287.99)<br>a	175,64<br>(161.76 - 194.11)<br>b	0,00<br>(0.00 - 0.00)<br>c	II
**2-Methylpropanal**	351,26<br>(328.44 - 366.31)<br>cd	461,27<br>(444.88 - 473.13)<br>c	36,33<br>(28.35 - 44.28)<br>f	75,03<br>(61.80 - 99.07)<br>ef	76,62<br>(59.03 - 88.38)<br>ef	0,00<br>(0.00 - 0.00)<br>f	202,43<br>(172.00 - 255.19)<br>de	173,63<br>(154.36 - 216.52)<br>def	81,47<br>(72.48 - 112.11)<br>def	0,00<br>(0.00 - 0.00)<br>f	0,00<br>(0.00 - 0.00)<br>f	68,84<br>(59.41 - 79.61)<br>ef	1.100,89<br>(1,026.72 - 1,438.86)<br>b	1.517,75<br>(1,253.39 - 1,749.88)<br>a	1.206,01<br>(1,018.35 - 1,436.02)<br>b	0,00<br>(0.00 - 0.00)<br>f	II
**3-Methylbutanal**	16,50<br>(13.06 - 17.72)<br>d	10,74<br>(9.49 - 11.25)<br>d	6,47<br>(5.67 - 7.36)<br>d	3,08<br>(2.68 - 3.32)<br>d	31,75<br>(24.21 - 44.39)<br>d	2,47<br>(2.12 - 2.60)<br>d	96,15<br>(88.07 - 133.82)<br>c	129,52<br>(124.78 - 135.93)<br>c	26,24<br>(25.72 - 29.04)<br>d	0,00<br>(0.00 - 0.00)<br>d	0,00<br>(0.00 - 0.00)<br>d	25,50<br>(21.67 - 26.98)<br>d	375,70<br>(344.79 - 529.81)<br>b	499,10<br>(473.66 - 618.22)<br>a	502,46<br>(462.47 - 565.95)<br>a	0,00<br>(0.00 - 0.00)<br>d	II
**2-Methylbutanal**	44,39<br>(34.92 - 46.11)<br>cde	29,40<br>(29.09 - 30.07)<br>de	14,69<br>(13.64 - 16.63)<br>e	7,79<br>(6.94 - 9.13)<br>e	102,94<br>(85.91 - 122.28)<br>cde	6,09<br>(5.85 - 7.36)<br>e	182,96<br>(172.25 - 260.56)<br>c	201,64<br>(165.54 - 214.36)<br>cd	99,39<br>(91.40 - 123.06)<br>cde	3,77<br>(0.00 - 4.08)<br>e	0,00<br>(0.00 - 0.00)<br>e	98,35<br>(80.14 - 116.70)<br>cde	828,89<br>(763.02 - 1,142.87)<br>b	1.151,38<br>(1,021.66 - 1,382.78)<br>a	1.133,50<br>(1,034.97 - 1,268.89)<br>a	0,00<br>(0.00 - 0.00)<br>e	II
**Pentanal**	0,00<br>(0.00 - 0.00)<br>c	0,00<br>(0.00 - 0.00)<br>c	0,00<br>(0.00 - 0.00)<br>c	0,00<br>(0.00 - 0.00)<br>c	0,00<br>(0.00 - 0.00)<br>c	0,00<br>(0.00 - 0.00)<br>c	0,00<br>(0.00 - 0.00)<br>c	0,00<br>(0.00 - 0.00)<br>c	0,00<br>(0.00 - 0.00)<br>c	0,00<br>(0.00 - 0.00)<br>c	0,00<br>(0.00 - 0.00)<br>c	0,00<br>(0.00 - 0.00)<br>c	52,31<br>(47.37 - 72.82)<br>b	92,20<br>(84.41 - 151.21)<br>a	113,65<br>(109.55 - 139.83)<br>a	0,00<br>(0.00 - 0.00)<br>c	II
**Hexanal**	0,00<br>(0.00 - 0.00)<br>c	0,00<br>(0.00 - 0.00)<br>c	0,00<br>(0.00 - 0.00)<br>c	0,00<br>(0.00 - 0.00)<br>c	0,00<br>(0.00 - 0.00)<br>c	0,00<br>(0.00 - 0.00)<br>c	0,00<br>(0.00 - 0.00)<br>c	0,00<br>(0.00 - 0.00)<br>c	0,00<br>(0.00 - 0.00)<br>c	0,00<br>(0.00 - 0.00)<br>c	0,00<br>(0.00 - 0.00)<br>c	0,00<br>(0.00 - 0.00)<br>c	500,41<br>(455.85 - 707.29)<br>b	840,49<br>(741.36 - 1,138.34)<br>a	833,30<br>(759.45 - 1,007.38)<br>a	0,00<br>(0.00 - 0.00)<br>c	II
**Heptanal**	0,00<br>(0.00 - 0.00)<br>c	0,00<br>(0.00 - 0.00)<br>c	0,00<br>(0.00 - 0.00)<br>c	0,00<br>(0.00 - 0.00)<br>c	0,00<br>(0.00 - 0.00)<br>c	0,00<br>(0.00 - 0.00)<br>c	0,00<br>(0.00 - 0.00)<br>c	0,00<br>(0.00 - 0.00)<br>c	0,00<br>(0.00 - 0.00)<br>c	0,00<br>(0.00 - 0.00)<br>c	0,00<br>(0.00 - 0.00)<br>c	0,00<br>(0.00 - 0.00)<br>c	4,68<br>(4.04 - 5.67)<br>b	6,98<br>(6.10 - 15.26)<br>a	7,91<br>(7.53 - 9.66)<br>a	0,00<br>(0.00 - 0.00)<br>c	II
**Pentane**	44.556,69<br>(42,589.77 - 52,807.24)<br>b	61.477,87<br>(54,548.80 - 65,871.91)<br>a	11.193,15<br>(9,766.73 - 12,767.83)<br>f	10.132,78<br>(9,627.52 - 12,320.57)<br>f	38.342,70<br>(34,398.85 - 42,808.96)<br>bc	46.496,79<br>(44,560.68 - 50,039.49)<br>b	26.857,52<br>(20,812.87 - 30,151.86)<br>e	37.553,64<br>(35,703.16 - 38,177.82)<br>bc	39.723,65<br>(35,420.79 - 45,182.91)<br>bc	27.900,14<br>(23,291.91 - 32,349.55)<br>de	11.219,66<br>(10,839.22 - 12,575.30)<br>f	34.022,76<br>(31,268.06 - 37,792.23)<br>cd	421,22<br>(259.08 - 654.57)<br>g	663,73<br>(515.05 - 791.16)<br>g	633,58<br>(507.49 - 779.26)<br>g	0,00<br>(0.00 - 0.00)<br>g	Ia
**Heptane**	32,89<br>(31.96 - 43.81)<br>cde	64,06<br>(54.70 - 74.34)<br>a	17,02<br>(15.77 - 19.40)<br>efg	14,07<br>(12.53 - 15.95)<br>fgh	74,22<br>(66.12 - 84.48)<br>a	55,04<br>(43.16 - 70.76)<br>abc	38,72<br>(32.03 - 43.22)<br>cd	46,93<br>(43.82 - 48.91)<br>bc	56,28<br>(50.29 - 74.54)<br>ab	24,92<br>(23.47 - 27.33)<br>def	18,69<br>(17.77 - 19.67)<br>efg	52,04<br>(50.97 - 54.42)<br>bc	0,00<br>(0.00 - 0.00)<br>gh	0,00<br>(0.00 - 0.00)<br>h	0,00<br>(0.00 - 0.00)<br>h	0,00<br>(0.00 - 0.00)<br>h	Ib
**Octane**	15,78<br>(14.52 - 21.05)<br>def	26,00<br>(24.08 - 44.28)<br>bc	9,12<br>(8.20 - 10.02)<br>f	6,69<br>(5.87 - 7.87)<br>fg	44,05<br>(41.99 - 50.07)<br>a	38,69<br>(27.37 - 42.14)<br>b	25,10<br>(22.77 - 28.06)<br>cde	27,82<br>(26.42 - 32.45)<br>bcd	39,57<br>(31.18 - 44.61)<br>b	16,65<br>(15.96 - 18.19)<br>ef	10,19<br>(9.42 - 11.84)<br>f	32,78<br>(25.73 - 34.16)<br>bcd	0,00<br>(0.00 - 0.15)<br>g	0,00<br>(0.00 - 0.77)<br>g	0,00<br>(0.00 - 0.00)<br>g	0,00<br>(0.00 - 0.00)<br>g	Ib
**Nonane**	0,68<br>(0.59 - 0.78)<br>b	0,92<br>(0.80 - 1.05)<br>a	0,00<br>(0.00 - 0.22)<br>g	0,00<br>(0.00 - 0.00)<br>g	0,43<br>(0.39 - 0.50)<br>cd	0,41<br>(0.34 - 0.50)<br>cde	0,25<br>(0.00 - 0.27)<br>fg	0,27<br>(0.25 - 0.28)<br>ef	0,55<br>(0.39 - 0.63)<br>bc	0,00<br>(0.00 - 0.00)<br>g	0,00<br>(0.00 - 0.25)<br>g	0,35<br>(0.33 - 0.37)<br>de	0,00<br>(0.00 - 0.00)<br>g	0,00<br>(0.00 - 0.00)<br>g	0,00<br>(0.00 - 0.00)<br>g	0,00<br>(0.00 - 0.00)<br>g	Ib
**Hexane**	34,84<br>(33.07 - 36.81)<br>cd	48,90<br>(47.06 - 53.19)<br>ab	17,61<br>(16.30 - 19.66)<br>ef	13,43<br>(12.22 - 17.67)<br>ef	48,08<br>(39.79 - 57.71)<br>abc	54,00<br>(43.96 - 59.16)<br>a	41,47<br>(37.88 - 41.71)<br>c	41,89<br>(39.59 - 42.57)<br>bc	50,18<br>(41.10 - 52.19)<br>abc	22,49<br>(20.80 - 25.27)<br>de	16,67<br>(13.66 - 19.07)<br>ef	45,93<br>(41.29 - 46.75)<br>abc	11,51<br>(8.49 - 14.35)<br>ef	17,93<br>(11.24 - 18.56)<br>ef	10,36<br>(9.51 - 10.56)<br>fg	0,00<br>(0.00 - 0.00)<br>g	Ia
**2,2-Dimethylbutane**	40,52<br>(40.21 - 49.64)<br>ab	88,11<br>(60.19 - 108.35)<br>a	12,01<br>(9.86 - 31.35)<br>bc	35,38<br>(10.20 - 48.67)<br>b	24,94<br>(17.99 - 35.95)<br>b	25,57<br>(23.18 - 29.98)<br>bc	23,83<br>(22.99 - 25.49)<br>bc	23,40<br>(21.61 - 24.01)<br>bc	25,59<br>(25.15 - 59.80)<br>b	13,85<br>(12.27 - 36.96)<br>bc	16,24<br>(14.56 - 47.00)<br>bc	18,00<br>(16.45 - 21.48)<br>bc	13,85<br>(12.74 - 18.27)<br>bc	19,36<br>(16.46 - 20.93)<br>bc	28,26<br>(12.27 - 43.73)<br>bc	0,00<br>(0.00 - 0.00)<br>c	Ia
**2,3-Dimethylbutane**	0,73<br>(0.61 - 0.96)<br>abc	2,28<br>(1.57 - 2.84)<br>a	0,48<br>(0.35 - 1.44)<br>abc	1,32<br>(0.35 - 1.79)<br>ab	0,87<br>(0.34 - 1.19)<br>abc	1,03<br>(0.69 - 1.20)<br>abc	0,81<br>(0.61 - 0.97)<br>abc	0,74<br>(0.64 - 1.13)<br>abc	0,91<br>(0.84 - 2.47)<br>ab	0,34<br>(0.29 - 1.15)<br>bc	1,15<br>(0.45 - 1.57)<br>abc	0,55<br>(0.50 - 0.69)<br>bc	0,44<br>(0.37 - 0.69)<br>bc	0,68<br>(0.41 - 0.78)<br>bc	0,90<br>(0.36 - 1.36)<br>abc	0,00<br>(0.00 - 0.00)<br>c	Ia
**2-Methylpentane**	286,65<br>(254.51 - 293.16)<br>ab	389,24<br>(284.88 - 412.84)<br>a	136,81<br>(85.93 - 193.66)<br>bc	156,33<br>(66.44 - 248.69)<br>bc	221,50<br>(172.07 - 274.89)<br>ab	178,07<br>(149.68 - 227.91)<br>ab	0,00<br>(0.00 - 0.00)<br>c	118,54<br>(103.57 - 148.40)<br>bc	252,39<br>(228.75 - 445.65)<br>a	178,10<br>(158.81 - 293.31)<br>ab	130,32<br>(85.41 - 262.01)<br>abc	157,09<br>(135.45 - 202.32)<br>ab	91,41<br>(84.36 - 137.54)<br>bc	128,14<br>(109.49 - 147.50)<br>bc	157,89<br>(77.24 - 253.76)<br>abc	0,00<br>(0.00 - 0.00)<br>c	III
**3-Methylpentane**	62,55<br>(58.70 - 71.29)<br>abc	111,34<br>(76.71 - 125.80)<br>a	29,16<br>(23.81 - 43.37)<br>cd	46,02<br>(25.97 - 59.59)<br>c	53,07<br>(46.15 - 74.31)<br>bc	63,11<br>(48.27 - 68.48)<br>abc	67,81<br>(56.52 - 69.20)<br>abc	54,65<br>(51.46 - 56.37)<br>bc	73,03<br>(53.66 - 96.10)<br>ab	26,30<br>(23.41 - 49.59)<br>cd	25,79<br>(22.24 - 51.55)<br>cd	49,42<br>(42.55 - 50.81)<br>bc	31,00<br>(25.21 - 43.60)<br>cd	49,90<br>(41.27 - 51.67)<br>bc	41,58<br>(27.98 - 55.89)<br>cd	0,00<br>(0.00 - 0.00)<br>d	III
**Methylcyclo-pentane**	5,17<br>(4.26 - 5.20)<br>def	5,28<br>(4.99 - 5.49)<br>cde	3,15<br>(2.89 - 3.75)<br>ef	2,50<br>(2.07 - 3.58)<br>f	6,81<br>(5.75 - 8.12)<br>bc	6,36<br>(5.83 - 8.15)<br>ab	9,29<br>(7.80 - 9.68)<br>a	6,33<br>(5.85 - 6.48)<br>bcd	8,27<br>(6.09 - 8.49)<br>ab	2,81<br>(2.71 - 3.10)<br>f	2,75<br>(2.24 - 2.96)<br>f	6,59<br>(5.81 - 7.01)<br>bcd	3,44<br>(2.66 - 3.89)<br>ef	5,73<br>(3.55 - 5.85)<br>de	2,89<br>(2.68 - 3.00)<br>f	0,00<br>(0.00 - 0.00)<br>g	III

median<br>(percentiles)<br>Tukey-HSD-test	*M. bovis*	*M. avium* ssp. *paratuberculosis*	*M. avium* ssp. *avium*	*M. avium* ssp. *hominissuis*	*M. intracellulare*	*M. terrae*	*M. marinum* incubated at 37°C	*M. marinum* incubated at 30°C	*M. diernhoferi*	*M. fortuitum*	*M. phlei*	*M. smegmatis*	control vials incubated for 2 weeks	control vials incubated for 3 weeks	control vials incubated for 4 weeks	laboratory air	group
**Benzaldehyde**	0,00<br>(0.00 - 0.00)<br>c	0,00<br>(0.00 - 0.00)<br>c	0,00<br>(0.00 - 0.00)<br>c	0,00<br>(0.00 - 0.00)<br>c	0,00<br>(0.00 - 0.00)<br>c	0,00<br>(0.00 - 0.00)<br>c	0,00<br>(0.00 - 0.00)<br>c	0,00<br>(0.00 - 0.00)<br>c	0,00<br>(0.00 - 0.00)<br>c	0,00<br>(0.00 - 0.00)<br>c	0,00<br>(0.00 - 0.00)<br>c	0,00<br>(0.00 - 0.00)<br>c	31,06<br>(23.59 - 34.19)<br>b	38,43<br>(35.23 - 64.46)<br>a	42,70<br>(38.82 - 55.51)<br>a	0,00<br>(0.00 - 0.00)<br>c	II
**2-Methyl-propionic acid ME**	4,51<br>(4.28 - 4.52)<br>bc	4,94<br>(4.65 - 5.79)<br>b	18,36<br>(15.48 - 20.48)<br>a	6,82<br>(5.39 - 7.37)<br>b	0,00<br>(0.00 - 0.60)<br>e	1,01<br>(0.96 - 1.40)<br>cde	7,45<br>(5.75 - 7.97)<br>b	2,50<br>(2.30 - 2.58)<br>cd	0,54<br>(0.00 - 0.70)<br>de	0,62<br>(0.57 - 0.70)<br>de	0,00<br>(0.00 - 0.00)<br>e	0,58<br>(0.48 - 0.68)<br>de	0,00<br>(0.00 - 0.00)<br>e	0,00<br>(0.00 - 0.00)<br>e	0,00<br>(0.00 - 0.00)<br>e	0,00<br>(0.00 - 0.00)<br>e	Ib
**3-Methyl-1-butanol acetate**	0,00<br>(0.00 - 0.00)<br>d	0,00<br>(0.00 - 0.00)<br>d	0,00<br>(0.00 - 0.00)<br>d	0,00<br>(0.00 - 0.00)<br>d	0,99<br>(0.92 - 1.07)<br>b	0,00<br>(0.00 - 0.00)<br>d	0,76<br>(0.64 - 0.89)<br>bc	0,58<br>(0.50 - 0.60)<br>c	1,31<br>(1.13 - 1.73)<br>a	0,00<br>(0.00 - 0.00)<br>d	0,00<br>(0.00 - 0.00)<br>d	0,87<br>(0.83 - 0.91)<br>b	0,00<br>(0.00 - 0.00)<br>d	0,00<br>(0.00 - 0.00)<br>d	0,00<br>(0.00 - 0.00)<br>d	0,00<br>(0.00 - 0.00)<br>d	Ib
**Benzioc acid ME**	0,00<br>(0.00 - 0.00)<br>b	0,00<br>(0.00 - 0.00)<br>b	8,08<br>(6.46 - 10.07)<br>a	0,00<br>(0.00 - 1.48)<br>b	0,00<br>(0.00 - 0.00)<br>b	0,00<br>(0.00 - 0.00)<br>b	1,33<br>(0.00 - 1.45)<br>b	0,00<br>(0.00 - 0.00)<br>b	0,00<br>(0.00 - 0.00)<br>b	0,00<br>(0.00 - 0.00)<br>b	0,00<br>(0.00 - 0.00)<br>b	0,00<br>(0.00 - 0.00)<br>b	0,00<br>(0.00 - 0.00)<br>b	0,00<br>(0.00 - 0.00)<br>b	0,00<br>(0.00 - 0.00)<br>b	0,00<br>(0.00 - 0.00)<br>b	Ib
**Furan**	34,47<br>(29.54 - 42.30)<br>de	86,45<br>(52.06 - 104.98)<br>de	133,89<br>(93.69 - 201.17)<br>de	130,54<br>(97.84 - 142.15)<br>de	206,30<br>(164.97 - 293.66)<br>bc	231,08<br>(188.55 - 258.23)<br>bcd	412,06<br>(386.23 - 422.10)<br>a	356,62<br>(334.01 - 374.21)<br>ab	372,52<br>(339.56 - 497.10)<br>a	170,69<br>(133.24 - 181.49)<br>cd	139,41<br>(107.72 - 149.95)<br>cde	219,15<br>(215.22 - 309.46)<br>bc	257,06<br>(227.04 - 317.89)<br>abc	349,72<br>(270.61 - 356.87)<br>ab	217,72<br>(204.99 - 245.91)<br>bcd	0,00<br>(0.00 - 0.00)<br>e	III
**2-Methylfuran**	166,21<br>(130.89 - 172.06)<br>ef	369,40<br>(355.19 - 408.03)<br>a	59,31<br>(46.84 - 78.09)<br>g	36,69<br>(33.75 - 53.54)<br>gh	66,64<br>(60.03 - 76.50)<br>g	62,99<br>(57.49 - 66.33)<br>g	229,04<br>(227.38 - 263.39)<br>cd	66,43<br>(64.00 - 69.07)<br>g	85,98<br>(74.35 - 104.20)<br>fg	31,99<br>(30.14 - 33.79)<br>gh	31,52<br>(28.61 - 37.37)<br>gh	62,29<br>(60.90 - 71.87)<br>g	182,27<br>(149.30 - 243.31)<br>de	361,04<br>(262.72 - 407.15)<br>ab	285,84<br>(253.12 - 351.69)<br>bc	0,00<br>(0.00 - 0.00)<br>h	III
**2-Ethylfuran**	215,53<br>(163.90 - 222.06)<br>cd	494,44<br>(445.67 - 524.13)<br>a	82,27<br>(67.00 - 100.30)<br>e	39,06<br>(34.70 - 47.25)<br>ef	77,64<br>(72.26 - 82.03)<br>e	62,87<br>(56.79 - 68.60)<br>ef	275,36<br>(252.24 - 289.76)<br>c	50,17<br>(49.10 - 52.55)<br>ef	98,45<br>(78.30 - 103.98)<br>de	41,01<br>(35.67 - 42.01)<br>ef	63,94<br>(60.94 - 76.62)<br>ef	72,21<br>(69.28 - 85.64)<br>e	178,35<br>(154.88 - 246.55)<br>c	355,38<br>(334.04 - 460.62)<br>b	401,31<br>(382.30 - 469.47)<br>ab	0,00<br>(0.00 - 0.00)<br>f	II
**2-Propylfuran**	1,32<br>(1.16 - 1.40)<br>de	4,99<br>(4.68 - 5.97)<br>a	0,00<br>(0.00 - 0.00)<br>fg	0,00<br>(0.00 - 0.00)<br>g	0,00<br>(0.00 - 0.00)<br>fg	0,00<br>(0.00 - 0.00)<br>g	1,94<br>(1.82 - 2.11)<br>cd	0,00<br>(0.00 - 0.00)<br>g	0,86<br>(0.00 - 1.08)<br>ef	0,00<br>(0.00 - 0.00)<br>g	0,00<br>(0.00 - 0.00)<br>g	0,00<br>(0.00 - 0.00)<br>g	1,17<br>(0.99 - 1.63)<br>de	2,38<br>(2.22 - 3.79)<br>bc	2,91<br>(2.78 - 3.40)<br>b	0,00<br>(0.00 - 0.00)<br>g	III
**2,3,5-Trimethylfuran**	7,91<br>(7.51 - 8.31)<br>b	11,61<br>(10.81 - 12.67)<br>a	1,58<br>(1.41 - 2.07)<br>fgh	0,97<br>(0.92 - 1.36)<br>h	2,91<br>(2.51 - 3.28)<br>de	2,68<br>(2.59 - 2.85)<br>de	3,25<br>(2.68 - 3.96)<br>cd	1,56<br>(1.48 - 2.04)<br>fgh	3,76<br>(3.14 - 4.88)<br>c	1,30<br>(1.27 - 1.39)<br>gh	1,56<br>(1.48 - 1.74)<br>fgh	2,63<br>(2.34 - 2.80)<br>def	1,03<br>(1.01 - 1.56)<br>gh	1,93<br>(1.82 - 2.75)<br>efg	1,58<br>(1.48 - 1.80)<br>fgh	0,00<br>(0.00 - 0.00)<br>i	Ia
**2n-Butylfuran**	1,12<br>(1.09 - 1.25)<br>bc	4,65<br>(4.13 - 5.45)<br>a	0,00<br>(0.00 - 0.00)<br>e	0,00<br>(0.00 - 0.00)<br>e	0,00<br>(0.00 - 0.00)<br>e	0,00<br>(0.00 - 0.00)<br>e	1,05<br>(0.99 - 1.22)<br>c	0,00<br>(0.00 - 0.00)<br>e	0,39<br>(0.00 - 0.46)<br>de	0,00<br>(0.00 - 0.00)<br>e	0,00<br>(0.00 - 0.00)<br>e	0,00<br>(0.00 - 0.00)<br>e	0,69<br>(0.62 - 0.87)<br>cd	1,43<br>(1.32 - 2.31)<br>b	1,82<br>(1.65 - 2.18)<br>b	0,00<br>(0.00 - 0.00)<br>e	III
**Dibromochloro-methane**	0,22<br>(0.11 - 0.23)<br>h	0,22<br>(0.21 - 0.23)<br>gh	0,33<br>(0.32 - 0.36)<br>de	0,28<br>(0.26 - 0.30)<br>fg	0,37<br>(0.34 - 0.39)<br>cd	0,41<br>(0.38 - 0.43)<br>bcd	0,42<br>(0.40 - 0.42)<br>abc	0,45<br>(0.43 - 0.47)<br>ab	0,42<br>(0.36 - 0.46)<br>abcd	0,33<br>(0.32 - 0.36)<br>def	0,28<br>(0.27 - 0.30)<br>efg	0,38<br>(0.35 - 0.40)<br>bcd	0,44<br>(0.41 - 0.47)<br>ab	0,45<br>(0.43 - 0.51)<br>a	0,38<br>(0.34 - 0.45)<br>abcd	0,00<br>(0.00 - 0.00)<br>i	III
**Acetone**	20.048,28<br>(18,003.51 - 20,940.16)<br>a	13.025,55<br>(11,918.44 - 14,504.16)<br>bc	4.438,55<br>(3,444.20 - 5,069.92)<br>fg	5.464,46<br>(4,692.71 - 5,812.86)<br>ef	2.487,47<br>(2,202.68 - 3,279.33)<br>gh	12.180,60<br>(10,007.23 - 15,352.66)<br>c	14.398,24<br>(13,227.80 - 15,355.27)<br>b	9.189,58<br>(8,625.12 - 9,286.64)<br>d	7.118,28<br>(6,681.13 - 8,074.54)<br>de	8.904,87<br>(7,347.51 - 9,793.40)<br>d	4.423,48<br>(3,525.74 - 4,674.48)<br>fgh	2.697,50<br>(2,385.47 - 2,745.04)<br>gh	1.927,90<br>(1,523.13 - 2,039.56)<br>hi	2.549,20<br>(2,361.59 - 3,101.23)<br>gh	2.293,62<br>(2,105.59 - 2,479.01)<br>ghi	0,00<br>(0.00 - 0.00)<br>i	Ia
**2,3-Butadione**	194,57<br>(159.08 - 196.85)<br>de	204,83<br>(190.53 - 213.57)<br>d	57,47<br>(40.71 - 78.15)<br>de	61,45<br>(51.52 - 101.47)<br>de	35,01<br>(30.02 - 39.35)<br>e	1.298,39<br>(1,200.76 - 1,448.54)<br>a	674,47<br>(610.03 - 750.74)<br>c	68,64<br>(54.72 - 85.36)<br>de	28,54<br>(27.20 - 48.13)<br>e	878,35<br>(859.10 - 1,057.82)<br>b	142,74<br>(108.91 - 178.68)<br>de	17,93<br>(13.90 - 19.60)<br>e	5,48<br>(4.86 - 6.97)<br>e	5,78<br>(5.39 - 6.99)<br>e	5,20<br>(3.97 - 5.51)<br>e	0,00<br>(0.00 - 0.00)<br>e	Ia
**2-Butanone**	354,60<br>(353.80 - 367.38)<br>cd	402,35<br>(385.01 - 420.02)<br>c	266,83<br>(237.37 - 293.25)<br>d	171,52<br>(156.15 - 189.09)<br>e	291,22<br>(271.42 - 306.35)<br>d	418,58<br>(355.41 - 445.78)<br>c	956,97<br>(773.95 - 989.13)<br>a	513,82<br>(506.59 - 534.82)<br>b	343,71<br>(294.44 - 362.06)<br>cd	318,36<br>(287.68 - 384.47)<br>cd	140,15<br>(133.58 - 142.14)<br>e	259,18<br>(254.01 - 271.73)<br>d	116,60<br>(102.65 - 147.99)<br>e	153,68<br>(132.67 - 197.16)<br>e	142,03<br>(125.92 - 169.95)<br>e	0,00<br>(0.00 - 0.00)<br>f	Ia
**2-Pentanone**	19,31<br>(15.66 - 19.52)<br>cd	14,84<br>(14.74 - 16.31)<br>d	9,58<br>(7.80 - 11.42)<br>def	5,79<br>(5.28 - 6.76)<br>fgh	9,74<br>(8.44 - 10.91)<br>def	21,81<br>(17.95 - 27.03)<br>bc	65,74<br>(54.07 - 70.80)<br>a	26,98<br>(25.39 - 30.73)<br>b	11,85<br>(10.63 - 14.61)<br>de	11,21<br>(9.06 - 11.42)<br>def	3,60<br>(3.49 - 3.81)<br>gh	8,71<br>(8.32 - 9.20)<br>defg	3,51<br>(2.82 - 4.11)<br>gh	5,26<br>(4.53 - 8.05)<br>fgh	6,12<br>(5.42 - 7.82)<br>efgh	0,00<br>(0.00 - 0.00)<br>h	Ia
**3-Pentanone**	72,32<br>(69.56 - 78.26)<br>b	104,02<br>(92.59 - 110.60)<br>a	39,70<br>(30.15 - 52.66)<br>c	18,78<br>(17.00 - 21.32)<br>de	15,32<br>(12.36 - 16.74)<br>ef	20,39<br>(17.69 - 22.64)<br>de	43,18<br>(42.51 - 46.50)<br>c	22,54<br>(21.18 - 24.62)<br>d	13,99<br>(11.48 - 15.21)<br>ef	9,33<br>(8.10 - 10.00)<br>fg	10,87<br>(10.78 - 12.33)<br>ef	10,46<br>(9.40 - 11.62)<br>efg	1,20<br>(1.02 - 1.99)<br>gh	1,74<br>(1.54 - 1.88)<br>gh	1,66<br>(1.47 - 1.69)<br>gh	0,00<br>(0.00 - 0.00)<br>h	Ia
**Methylisobutyl-ketone**	4,45<br>(4.26 - 4.80)<br>bc	4,09<br>(3.54 - 4.48)<br>c	2,47<br>(2.08 - 3.20)<br>c	3,09<br>(2.50 - 3.48)<br>c	166,58<br>(158.95 - 193.91)<br>a	35,08<br>(29.88 - 42.66)<br>bc	26,80<br>(24.77 - 28.59)<br>bc	21,13<br>(19.70 - 25.08)<br>bc	195,86<br>(190.21 - 230.49)<br>a	14,64<br>(13.04 - 15.96)<br>bc	4,16<br>(3.72 - 4.72)<br>c	28,82<br>(21.24 - 41.04)<br>bc	1,53<br>(1.24 - 1.92)<br>bc	3,05<br>(2.06 - 120.38)<br>b	1,82<br>(1.69 - 1.95)<br>c	0,00<br>(0.00 - 0.00)<br>c	Ia
**2-Heptanone**	3,61<br>(3.37 - 3.83)<br>cde	4,27<br>(3.84 - 4.56)<br>c	0,86<br>(0.00 - 0.98)<br>f	0,91<br>(0.78 - 1.03)<br>f	1,40<br>(1.21 - 1.53)<br>ef	3,47<br>(3.27 - 4.08)<br>cd	3,60<br>(2.30 - 4.10)<br>cde	0,98<br>(0.89 - 1.03)<br>ef	1,33<br>(1.10 - 1.54)<br>ef	1,90<br>(1.59 - 1.91)<br>def	1,00<br>(0.89 - 1.11)<br>ef	0,88<br>(0.81 - 0.96)<br>f	2,87<br>(2.73 - 3.81)<br>cde	6,00<br>(5.47 - 11.32)<br>b	9,08<br>(8.09 - 12.18)<br>a	0,00<br>(0.00 - 0.00)<br>f	II
**3-Octanone**	10,51<br>(10.21 - 13.04)<br>bc	16,23<br>(16.05 - 19.20)<br>ab	3,28<br>(2.97 - 3.92)<br>ef	2,94<br>(2.67 - 4.13)<br>ef	9,82<br>(9.09 - 11.66)<br>c	5,14<br>(3.23 - 6.23)<br>def	22,23<br>(10.57 - 24.25)<br>a	5,43<br>(5.05 - 6.46)<br>de	8,44<br>(6.73 - 9.52)<br>cd	1,20<br>(1.15 - 1.29)<br>fg	9,23<br>(7.01 - 9.43)<br>cd	5,91<br>(4.60 - 7.81)<br>de	0,00<br>(0.00 - 0.00)<br>g	0,00<br>(0.00 - 0.00)<br>g	0,00<br>(0.00 - 0.00)<br>g	0,00<br>(0.00 - 0.00)<br>g	Ib
**Acetonitrile**	126,40<br>(124.20 - 127.31)<br>abc	111,54<br>(43.46 - 146.83)<br>abc	53,81<br>(28.60 - 74.78)<br>cd	53,73<br>(40.48 - 63.70)<br>cd	51,44<br>(34.37 - 130.55)<br>bc	107,23<br>(85.95 - 125.09)<br>ab	137,96<br>(133.32 - 154.56)<br>a	109,85<br>(97.66 - 120.35)<br>abc	150,41<br>(145.18 - 153.26)<br>a	75,39<br>(37.83 - 85.48)<br>bcd	52,24<br>(21.44 - 62.61)<br>cd	88,53<br>(70.25 - 89.08)<br>abc	62,72<br>(33.38 - 67.08)<br>cd	69,59<br>(47.50 - 85.84)<br>bcd	39,51<br>(22.31 - 46.54)<br>cd	0,00<br>(0.00 - 0.00)<br>d	Ia
**Isobutyronitrile**	7,04<br>(6.42 - 8.11)<br>bcde	9,12<br>(7.73 - 10.61)<br>abcd	7,43<br>(6.63 - 7.78)<br>bcd	5,80<br>(5.49 - 6.84)<br>de	11,60<br>(10.87 - 12.58)<br>abc	10,66<br>(9.72 - 13.53)<br>abc	12,72<br>(11.49 - 14.37)<br>ab	11,86<br>(10.99 - 12.05)<br>abcd	13,18<br>(12.23 - 14.74)<br>a	6,90<br>(6.88 - 7.46)<br>bcde	5,25<br>(5.14 - 5.54)<br>de	10,22<br>(9.74 - 10.41)<br>a	6,05<br>(5.41 - 7.15)<br>cde	8,51<br>(6.34 - 10.26)<br>abcd	5,70<br>(5.37 - 6.84)<br>de	0,00<br>(0.00 - 0.00)<br>e	III
**2-Methylbutane-nitrile**	5,15<br>(4.69 - 5.43)<br>b	5,27<br>(4.67 - 5.88)<br>b	6,35<br>(5.83 - 7.22)<br>b	5,56<br>(4.85 - 6.39)<br>b	0,00<br>(0.00 - 0.00)<br>c	14,75<br>(14.61 - 18.65)<br>a	0,00<br>(0.00 - 0.00)<br>c	0,00<br>(0.00 - 0.00)<br>c	0,00<br>(0.00 - 0.00)<br>c	16,54<br>(15.57 - 17.24)<br>a	5,53<br>(4.84 - 5.67)<br>b	0,00<br>(0.00 - 0.00)<br>c	5,33<br>(4.09 - 5.93)<br>b	6,60<br>(5.87 - 8.50)<br>b	4,70<br>(4.35 - 5.48)<br>b	0,00<br>(0.00 - 0.00)<br>c	III
**3-Methylbutane-nitrile**	3,62<br>(3.29 - 3.99)<br>cd	4,11<br>(3.79 - 4.24)<br>cd	3,74<br>(3.53 - 4.43)<br>cd	2,92<br>(2.56 - 3.26)<br>d	6,14<br>(5.56 - 6.87)<br>ab	6,26<br>(5.23 - 7.25)<br>a	7,21<br>(6.98 - 7.49)<br>a	6,87<br>(6.01 - 7.12)<br>a	6,88<br>(5.70 - 7.52)<br>a	4,61<br>(4.32 - 4.86)<br>bc	3,17<br>(2.82 - 3.26)<br>cd	6,06<br>(5.39 - 7.31)<br>ab	3,30<br>(2.63 - 3.70)<br>cd	4,13<br>(3.35 - 5.52)<br>bc	2,90<br>(2.74 - 3.52)<br>cd	0,00<br>(0.00 - 0.00)<br>e	III
**Dimethyldisulfid**	22,94<br>(22.29 - 23.85)<br>abcd	26,78<br>(24.66 - 28.17)<br>ab	6,20<br>(5.83 - 7.17)<br>e	5,40<br>(4.66 - 6.51)<br>ef	23,10<br>(19.98 - 25.53)<br>bcd	0,92<br>(0.73 - 1.17)<br>efg	1,24<br>(0.00 - 1.91)<br>efg	0,41<br>(0.32 - 1.00)<br>efg	25,09<br>(20.70 - 30.02)<br>ab	0,00<br>(0.00 - 0.47)<br>fg	28,07<br>(25.62 - 32.59)<br>a	19,14<br>(17.87 - 19.60)<br>cd	14,59<br>(13.25 - 17.70)<br>d	21,90<br>(18.91 - 25.74)<br>abc	21,04<br>(20.43 - 23.33)<br>bcd	0,00<br>(0.00 - 0.00)<br>g	III
